# Novel Pyrrole Derivatives as Multi-Target Agents for the Treatment of Alzheimer’s Disease: Microwave-Assisted Synthesis, In Silico Studies and Biological Evaluation

**DOI:** 10.3390/ph17091171

**Published:** 2024-09-04

**Authors:** Emilio Mateev, Valentin Karatchobanov, Marjano Dedja, Konstantinos Diamantakos, Alexandrina Mateeva, Muhammed Tilahun Muhammed, Ali Irfan, Magdalena Kondeva-Burdina, Iva Valkova, Maya Georgieva, Alexander Zlatkov

**Affiliations:** 1 Department of Pharmaceutical Chemistry, Faculty of Pharmacy, Medical University, 1000 Sofia, Bulgaria; valentink1999@abv.bg (V.K.); mariosdedja@gmail.com (M.D.); konstantinosdiamantakos@gmail.com (K.D.); a.dineva@pharmfac.mu-sofia.bg (A.M.); mgeorgieva@pharmfac.mu-sofia.bg (M.G.); azlatkov@pharmfac.mu-sofia.bg (A.Z.); 2 Department of Pharmaceutical Chemistry, Faculty of Pharmacy, Suleyman Demirel University, 32260 Isparta, Türkiye; muhammedmuhammed@sdu.edu.tr; 3 Department of Chemistry, Government College University Faisalabad, Faisalabad 38000, Pakistan; raialiirfan@gmail.com; 4 Department of Pharmacology, Pharmacotherapy and Toxicology, Faculty of Pharmacy, Medical University, 1000 Sofia, Bulgaria; mkondeva@pharmfac.mu-sofia.bg; 5 Department of Chemistry, Faculty of Pharmacy, Medical University, 1000 Sofia, Bulgaria; ivalkova@pharmfac.mu-sofia.bg

**Keywords:** microwave-assisted synthesis, pyrrole, hydrazide–hydrazones, antioxidants, oxidative stress, molecular docking, DFT

## Abstract

Considering the complex pathogenesis of Alzheimer’s disease (AD), the multi-target ligand strategy is expected to provide superior effects for the treatment of the neurological disease compared to the classic single target strategy. Thus, one novel pyrrole-based hydrazide (**vh0**) and four corresponding hydrazide–hydrazones (**vh1-4**) were synthesized by applying highly efficient MW-assisted synthetic protocols. The synthetic pathway provided excellent yields and reduced reaction times under microwave conditions compared to conventional heating. The biological assays indicated that most of the novel pyrroles are selective MAO-B inhibitors with IC_50_ in the nanomolar range (665 nM) and moderate AChE inhibitors. The best dual-acting MAO-B/AChE inhibitor (IC_50_ *h*MAOB–0.665 μM; IC_50_ *ee*AChE—4.145 μM) was the unsubstituted pyrrole-based hydrazide (**vh0**). Importantly, none of the novel molecules displayed *h*MAOA-blocking capacities. The radical-scavenging properties of the compounds were examined using DPPH and ABTS in vitro tests. Notably, the hydrazide **vh0** demonstrated the best antioxidant activities. In addition, in silico simulations using molecular docking and MM/GBSA, targeting the AChE (PDB ID: **4EY6**) and MAO-B (PDB: **2V5Z**), were utilized to obtain active conformations and to optimize the most prominent dual inhibitor (**vh0**). The ADME and in vitro PAMPA studies demonstrated that **vh0** could cross the blood–brain barrier, and it poses good lead-like properties. Moreover, the optimized molecular structures and the frontier molecular orbitals were examined via DFT studies at 6-311G basis set in the ground state.

## 1. Introduction

Alzheimer’s disease (AD) is a chronic, progressive, neurodegenerative disorder associated with cognitive and behavioral alterations, accounting for more than half of dementia cases. Recent data indicated that, by 2050, its prevalence will double in Europe and triple worldwide [1]. There are many risk factors associated with AD including age, familial inheritance, exposure to aluminum, traumatic brain injuries and associated co-morbidities (vascular disease or infection). Early-onset familial AD form is rare and is associated with gene mutations [2]. The oxidative imbalance that leads to neuronal damage plays an important role in AD. AD is characterized by deposition of extracellular amyloid plaques (Aβ), intracellular tau (τ) protein aggregates and loss of synaptic links in regions of the main brain [3]. Promising pharmacological treatments are at a stage of advanced clinical trials and include anti-amyloid β and anti-tau strategies; however, several major drawbacks, such as high treatment cost with monoclonal antibodies, requirement for regular monitoring, and low efficacy of the treatment were discussed [4,5]. Therefore, the multi-target directed ligands (MTDLs) approach for the treatment of AD is one of the most promising alternatives [6]. Due to the involvement of monoamine oxidase B (MAO-B) in the formation of reactive oxygen species (ROS) and the association of high acetylcholinesterase (AChE) levels with learning and memory impairment, a common strategy is to design inhibitors against the former enzymes [7].

Importantly, the expression of MAO-B dominates in the human brain which makes it a viable target for the treatment of neurodegenerative conditions, such as Alzheimer’s and Parkinson’s diseases [8]. The former hypothesis is affirmed by the approval of two MAO-B inhibitors—Selegiline and Rasagiline—for the treatment of Parkinson’s disease. Moreover, the MAO-B-induced catalytic degradation of amines leads to the production of hydrogen peroxide (H_2_O_2_), which contributes to the formation of ROS. The formed 3,4-dihydroxyphenylacetaldehyde, ammonium molecule and H_2_O_2_ react with the iron ion (Fe^2+^) in dopaminergic neurons and hydroxyl radicals are formed which affects negatively the progression of AD [9].

Additionally, AChE inhibitors play a pivotal role in the maintenance of cholinergic functions and the former is frequently applied for symptomatic relief in AD [10]. The leading hypothesis of the pathogenesis of AD is the decrease in acetylcholine in the brain, which is the enhanced activity of AChE [11]. The active gorge of AChE comprises several subsites—catalytic, acyl pocket, oxyanion hole, anionic site and peripheral anionic site. The enzyme structure has a deep and narrow gorge, which could accommodate bulky chemical structures [12]. AChE is a viable therapeutic target for the treatment of AD—AChE inhibitors are one of the most commonly used medicines for AD patients [13,14].

The introduction of a pyrrole core motif in the new structures was prompted by the broad pharmacological profile of the former ring including antibacterial, anticonvulsant, antifungal, antiviral, anticancer, as well as antioxidant activities [15,16,17,18]. Moreover, several works defined the pyrrole moiety as a prominent pharmacophore feature in the design of novel MAO-B inhibitors [19,20]. Literature data concerning the potential of the hydrazide–hydrazone moiety against the MAO-B and AChE enzymes are also present [21,22]. Therefore, the interest in the implementation of the hydrazide–hydrazone moiety in pharmacologically active molecules is constantly increasing [23].

The field of microwave-assisted synthesis is growing rapidly considering the reduced reaction time, and drastically enhanced yields when compared to conventional heating [24]. The direct transfer of heat during the microwave synthesis completes the reactions several fold faster, with fewer unwanted side products [25]. Moreover, numerous studies report the environmentally friendly effect of the technique as it provides a possibility for a replacement of a toxic solvent with a green one [26,27].

Considering the broad pharmacological profiles of the pyrrole-based structures, the hydrazide–hydrazone fragment, as well as the reported advantages of the MW-assisted synthesis, the aim of this study was to implement both conventional and microwave-based approaches in the synthesis of a pyrrole-based hydrazide and the corresponding hydrazide–hydrazones. The newly reported compounds were assessed for their in vitro MAO-B/A, AChE, ABTS and DPPH activities. Aromatic side chain moieties bearing different substituents were selected to elucidate the binding affinity towards the aromatic cage of MAO-B. For a better understanding of the binding conformations and the energies of the molecular orbitals, molecular docking and DFT calculations were performed.

## 2. Results and Discussion

### 2.1. Design and Chemistry

**Figure 1:**
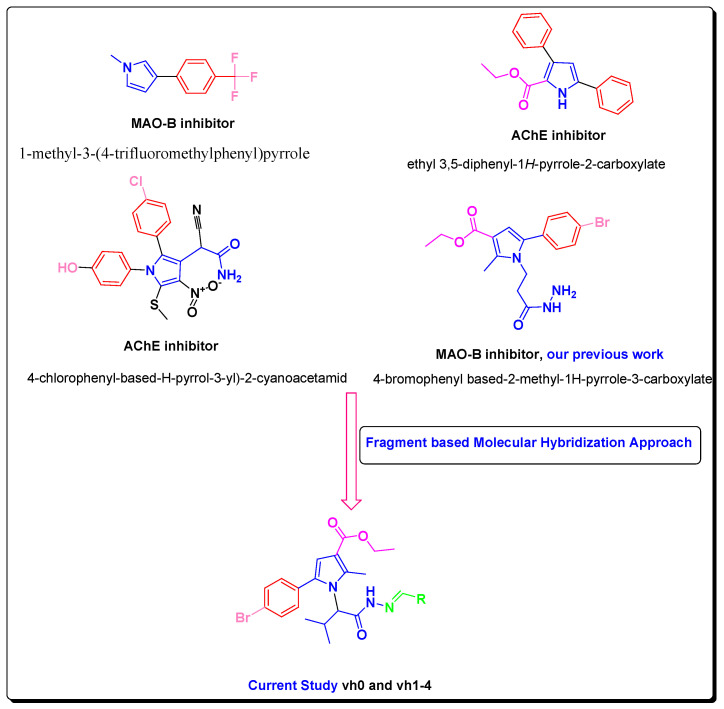
Rationale of newly designed compounds as AChE and MAO-B inhibitors.

In this study, we developed novel pyrrole-based compounds after initial Paal–Knorr condensation with the valine amino acid. To produce the targeted pyrrole derivatives, a fragment-based molecular hybridization approach was applied, as depicted in Figure 1 [19,28,29].

Besides the aforementioned pharmacological profile of the pyrrole derivatives, previously reported structure-based simulations demonstrated favorable hydrophobic interactions of the pyrrole ring with the active site of MAO-B, and orientation in the substrate pocket built up of the essential for the inhibitory capacity amino acids Phe168, Leu171, Ile199, and Tyr326 [8]. Importantly, the high selectivity of the novel compounds towards MAO-B is essential. That problem could be solved by altering the sizes of the new molecules. With a size of 550 Å^3^, the active cavity of MAO-A is more compact compared to the active gorge of MAO-B (700 Å^3^) [8]. The designed novel pyrrole-based molecules have a bulky character which hypothesizes possible selectivity towards MAO-B. The inhibition towards AChE could be initiated by designing bulky molecules that could interact with the peripheral cationic site (PAS) and/or the catalytic active site (CAS) of the crystal AChE. An introduction of a hydrazide–hydrazone moiety could lead to the formation of stable hydrogen bonds with the active sites of MAO-B and AChE [30]. The incorporation of a hydrazine (NH_2_-NH_2_) or a hydrazone (C=N−NH_2_) moiety could increase the radical scavenging properties of the new pyrrole-based compounds due to hydrogen atom transfer (HAT) and/or single electron transfer (SET) mechanisms.

**Scheme 1:**
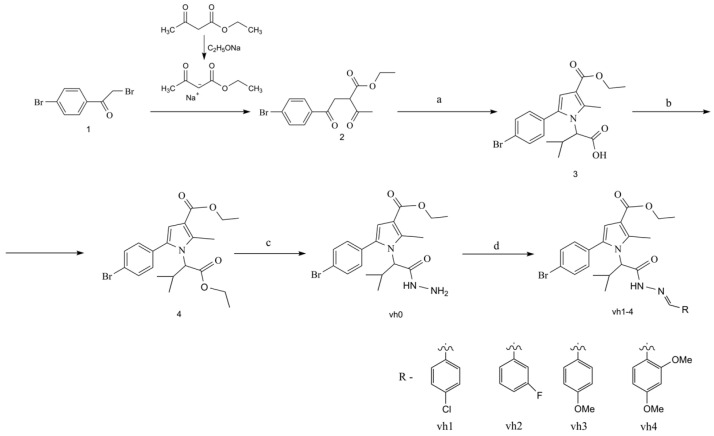
Synthesis of the compounds **vh0** and **vh1-4**. Reagents and reaction conditions: (**a**) valine, glacial acetic acid, MW reflux, 40 min.; (**b**) ethanol, thionyl chloride, MW reflux, 40 min.; (**c**) hydrazine hydrate, ab. ethanol, conventional (96 h) or MW (750 W, 1 h) reflux; (**d**) carbonyl compound (**1**–**4**), acetic acid, conventional reflux (30–50 min) or microwave heating (750 W, 1–3 min).

The synthetic approaches for the construction of the target pyrrole-based hydrazide–hydrazones (**vh1-vh4**) are illustrated in Scheme 1. The synthesis of the valine-based pyrrole **3** was achieved using the Paal–Knorr condensation, which involves the reaction of the dicarbonyl compound 2 and excess of the amino acid valine. Subsequently, the pyrrole-based acid was esterified by reacting it with thionyl chloride and absolute ethanol. The ethyl ester of the N-pyrrolylcarboxilic acid **4** was reacted with an excess of hydrazine-hydrate to obtain the novel hydrazide of the pyrrole **vh0**. According to the literature, an azomethine moiety is present in various MAO and AChE inhibitors [31,32]. Therefore, four novel hydrazide–hydrazones were produced after reacting the hydrazide vh0 with the carbonyl compounds in glacial acetic acid.

The novel compounds were confirmed by IR, ^1^H, ^13^C NMR and mass spectroscopy which are reported in the Section 3 of the work. The analytical and spectroscopic properties of all newly obtained compounds are in agreement with their expected chemical structures. The former data were summarized in the experimental section of the Supplementary Materials (Supplementary Figures S1–S15).

### 2.2. Microwave Synthesis

To reduce the reaction times and enhance the final yields of the hydrazide **vh0** and the title hydrazide–hydrazones **vh1-4**, microwave irradiation in a MW reactor was introduced. Microwave-assisted synthesis is one of the most prominent approaches for synthesizing various active compounds considering the uniform external energy, reduction of the reaction time, and high-yield possibilities compared to conventional heating. Moreover, green synthetic approaches are also considered using microwave-assisted synthesis [33]. Therefore, the syntheses of the hydrazide (**vh0**) and the hydrazide–hydrazones **vh1-4** were also carried out using MW irradiation. The microwave-assisted synthesis was carried out in a FlexiWave Milestone Lab reactor (Sorisole, Italy) equipped with a fiber optic and IR sensors.

The microwave-assisted synthesis of the reported pyrrole-based molecules provided higher yields (87–94%) when compared to the conventional method (55–85%). The reaction times and yields obtained for the hydrazide **vh0** and the Schiff bases **vh1-4** via both methods are summarized in Table 1.

The most significant reduction of the reaction time was observed during the hydrazinolysis of the N-pyrrolyl acid 4. The hydrazide **vh0** was formed after 96 h of reflux conditions when conventional heating was used. However, the MW heating led to a reaction time of 1 hr (FlexiWave Milestone Lab reactor, 750 W, Sorisole—Italy). The title hydrazide–hydrazones **vh1-4** were obtained after 1–3 min of MW irradiation (FlexiWave Milestone Lab reactor, 750 W, Sorisole—Italy). The displayed advantages of MW synthesis of hydrazide–hydrazones are in accordance with recently published works [23].

### 2.3. Antioxidant Activities

Two methods were used to observe the free-radical scavenging capacities of the title valine-based pyrroles—DPPH and ABTS assays.

### 2.4. DPPH Assay

The test observes the hydrogen donating capacity of the molecules which is shown with a decrease in the absorbance at 517 nm. The DPPH radical scavenging activity of the title hydrazide vh0 and hydrazide–hydrazones **vh1-4** was determined in 1 mg/mL concentration with Trolox applied as a standard.

The obtained results demonstrated that only the novel hydrazide **vh0** expressed statistically significant DPPH radical scavenging activity. The radical scavenging effect equaled 76.10% for the presented concentrations. In comparison, the DPPH radical scavenging activity of Trolox, which was used as a standard, was 90.07%. Interestingly, none of the four newly synthesized hydrazide–hydrazones showed any DPPH radical scavenging activity. The observed high activity of the valine-based hydrazide **vh0** could be due to the presence of a free hydrazine (NH–NH_2_) group, which is capable of being a hydrogen atom donor [34].

### 2.5. ABTS Assay

**Figure 2:**
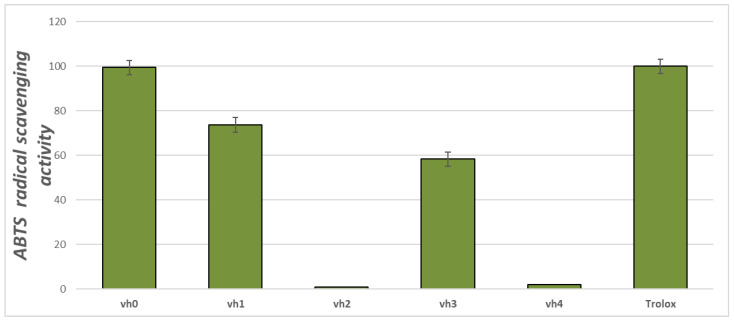
ABTS radical capacities of the title compounds (**vh0** and **vh1-4**) at concentrations of 1 mg/mL. Standard deviation (SD) (n = 3).

The ABTS test demonstrates the ability of the chemical compounds to neutralize the stable ABTS cation which could be observed at 734 nm. The ABTS antioxidant assay of the valine-based pyrroles is provided in Figure 2.

The obtained results indicated that three of the five newly synthesized compounds possessed ABTS radical-binding properties—**vh0**, **vh1** and **vh3**. The most pronounced ABTS radical-binding potential was demonstrated by **vh0**. Its activity at the applied concentration was 99.40%, comparable to that of the Trolox standard (100%). The ABTS radical-binding activity of **vh1** was 73.67% and that of **vh3** was 58.27%.

Overall, the compound with the greatest antioxidant potential was the valine-based hydrazide **vh0**. It could be hypothesized that the reported results are due to the presence of a free NH–NH_2_ group in its structure. The mono-substituted hydrazine group is capable of donating H-atoms from the amino group and thus binding free radicals [34]. In the hydrazide–hydrazones, the former moiety is blocked due to the presence of a fragment after a reaction with carbonyl compounds. Therefore, the radical-scavenging properties of the Schiff bases and their properties as donors of H-atoms are less pronounced. These data could be used for future design of antioxidant pyrrole-based compounds.

Importantly, the value of the radical-binding activity of the newly synthesized pyrrole-containing compounds was altered depending on the type of the applied test. Higher values of ABTS radical scavenging activity were observed compared to DPPH radical scavenging activity. Overall, the ABTS test was more suitable for the preliminary evaluation of the antioxidant potential of the newly synthesized compounds. The two main advantages of the ABTS test compared to the DPPH test are the ability to test both hydrophobic and hydrophilic molecules, since ABTS is a water-soluble molecule [35]. The second advantage is that the ABTS test allows the testing of bulky, high molecular weight molecules which is of importance in the particular case regarding the high molecular mass of the title compounds.

### 2.6. In Vitro MAO-B Evaluation

MAO inhibitors have a wide therapeutic range for the treatment of neurological and psychiatric conditions. The expression of type B MAO is increased in the hippocampus and cerebral cortex of the brains of patients suffering from AD in comparison to healthy brains. Moreover, enhanced degrees of active MAO-B enzymes are located in reactive astrocytes surrounding amyloid-β deposits [36]. The overexpression of MAO-B in astrocytes is theorized to catalyze the metabolism of monoamines and significantly increase the generation of free radicals and hydrogen peroxide (H_2_O_2_), thus MAO-B could be a proper target for the treatment of AD [9].

**Figure 3:**
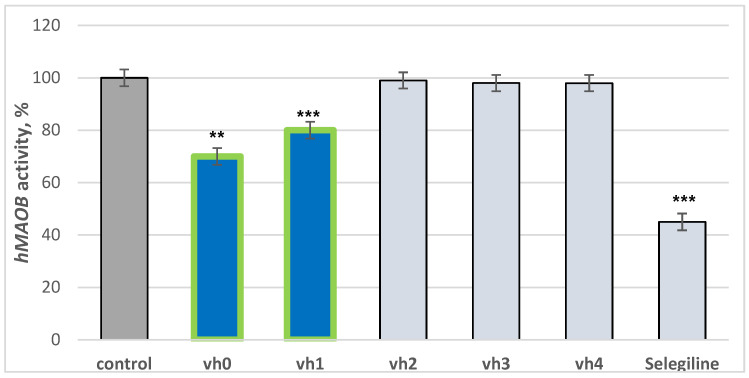
Inhibition capacity of the compounds **vh0-vh4** against *h*MAOB applied in 1 μM concentrations. Data are presented as means from three independent experiments ± SD. ** *p* < 0.01; *** *p* < 0.001 vs. control (pure *h*MAOB).

The title compounds were tested for *h*MAOB activities by an in vitro method. Selegiline was used as a reference drug. The results are provided in Figure 3. The novel pyrrole-based structures were tested in concentrations of 1 μM considering the IC_50_ of recently synthesized novel MAO-B inhibitors [9]. Elkamhawy et al. noted IC_50_ in the ranges of 0.66 to 2.41 μM, thus a 1 μM concentration was set in the current work.

The utilized standard selegiline decreased *h*MAOB activity by 55%, compared to the control (pure *h*MAOB). The hydrazide–hydrazone **vh0** demonstrated good activity of 30%. The examined activity of the hydrazide **vh0** is comparable to a recently reported study in which novel pyrrole derivatives displayed similar inhibition applied in 1 μM concentrations [37]. Out of all the hydrazide–hydrazones, only the compound condensed with 4-chlorobenzaldehyde—**vh1**—showed moderate activity towards *h*MAOB. Introducing 3-fluorine, 4-methoxy or 2,4-dimethoxy moieties in the aromatic benzene ring led to no MAO-B inhibition capacities. The introduction of a bulky benzene ring, substituted with different electron donating or electron withdrawing properties, lowered the MAO-B blocking effect. It could be hypothesized that the new fragments participate in steric clashes with the active site of the described enzyme.

### 2.7. In Vitro MAO-A Evaluation

The design and synthesis of selective MAO-B inhibitors is required considering the feasible chance of a hypertensive crisis triggered by an inhibition of MAO-A. The importance of selective MAO-B inhibitors in the treatment of neurodegenerative disorders was also discussed [8].

To observe the selectivity index of the title compounds, in vitro tests against MAO-A were carried out. The novel molecules were tested in the same concentrations as applied in the MAO-B evaluations—1 μM. Importantly, none of the tested compounds were able to inhibit MAO-A, which could be due to the smaller binding pocket of the isoenzyme. Both isoenzymes share more than 70% sequence identity; however, *h*MAOA’s active site consists of a single hydrophobic cavity with a size of approximately 555 Å^3^ which is more compact compared to the active gorge of the crystal *h*MAOB structure. Moreover, a “loop” conformation formed within the crystal *h*MAOA residues 210–216, differs from the *h*MAOB which could also affect the binding capacities [8].

### 2.8. In Vitro AChE Evaluation

One of the main causes leading to the development of AD is a decrease in the neurotransmitter acetylcholine (ACh) in the brain, mostly due to a higher activity of acetylcholinesterase (AChE). The design and synthesis of novel acetylcholinesterase inhibitors is the main option in the prevention of the progression of neurodegenerative disease [10].

**Figure 4:**
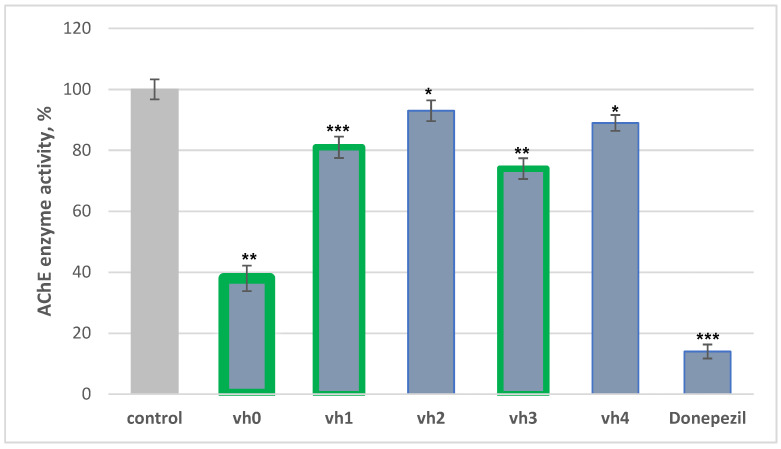
AChE assay of **vh0-4** applied as 10 μM concentrations. Data are presented as means from three independent experiments ± SD. * *p* < 0.1, ** *p* < 0.01, *** *p* < 0.001 vs. control (pure *ee*AChE).

The inhibitory capacity of the title compounds (**vh0** and **vh1-4**) against AChE (acetylcholinesterase) was evaluated according to the method of Ellman et al. with Donepezil as a reference compound [38]. Importantly, the novel compounds demonstrated good inhibitory effects in micromolar concentrations which is better compared to recent data [39]. The obtained results are provided in Figure 4.

The most active compound in our series was the unsubstituted hydrazide—**vh0**, with 62% activity against *ee*AChE. Two of the novel hydrazide–hydrazones demonstrated moderate inhibition of AChE—**vh1** with 19% and **vh3** with 26%. However, the hydrazide–hydrazone comprising 3-fluoro (**vh2**) and 2,4-dimethoxy (**vh3**) moieties showed weak AChE blocking activity. Overall, the placement of a substituted phenyl ring in the hydrazide moiety of **vh0** led to a decrease in the in vitro AChE activity, which could be due to steric hindrances.

### 2.9. Multi-Target Hit

Considering the reported problems surrounding the amyloid-based treatment for AD [8], as well as the not-so-promising paradigm “one drug, one target”, the multi-target directed ligands (MTDLs) approach remains one of the most frequently applied strategies [6]. Thus, the idea of AChE/MAO-B dual inhibitors has gained significant interest for the treatment of AD. Overall, the biological evaluations against MAO-A/B/AChE revealed that the pyrrole-based hydrazide—**vh0**, is a notable AChE/MAO-B inhibitor with high selectivity against the MAO type B. The dual-acting molecule showed good *h*MAOB (30% at 1 μM concentration) and excellent *ee*AChE-blocking capacity (62% at 10 μM concentration). For a more accessible comparison with the recently published data, the biological activities of the newly synthesized compounds were also reported as IC_50_ values (Table 2).

The presented results clearly demonstrate the multi-target effect of the novel hydrazide **vh0** exhibiting a half-inhibitory concentration (IC_50_) of 0.665 μM for MAO-B following a 2 h exposure and significant selective profile towards the former isoform. The latter value is comparable to the IC_50_ of the applied reference Selegiline (0.330 ± 0.09). The experimentally determined IC_50_ values toward AChE ranged from 4.145 for **vh0** to 16.262 for compound **vh3**. The Schiff bases **vh2** and **vh4** were inactive AChE inhibitors. The used standard Donepezil displayed better inhibitory properties with IC_50_ of 0.02.

The results obtained in this work displayed that unsubstituted pyrrole-based valine-containing derivatives possess the best dual-activating MAO-B and AChE inhibitory capacities. Moreover, it was noted that the former moieties lead to highly selective MAO inhibitors. When p-chlorobenzaldehyde was introduced into Schiff base formation, good dual capacities were observed.

Interestingly, another electron withdrawing fragment—3-fluoro moiety at the third position in the benzene ring—corresponded to no inhibitory activities. Furthermore, methoxy groups placed at 2- or 2,4- positions displayed a lack of inhibitory effect. Importantly, the unsubstituted hydrazide exerted the best dual-acting enzyme and selective (towards *h*MAOB) properties.

The described experimental results are better compared to recently published data for novel chalcone-based dual MAO-B/AChE inhibitors [40]. However, some optimized lead structures reported by Jeong et al. [41] showed inhibitory capacities in the nanomolar ranges. This leads to the necessity of further drug optimizations of **vh0**. In that regard, in silico simulations using molecular docking, MD, DFT, and ADME were carried out.

### 2.10. Molecular Docking

In an attempt to correlate the in vitro outcomes with in silico theoretical results and to gain further understanding of the active conformations of the novel structures, molecular docking simulations in the active sites of MAO-B (PDB: **2V5Z**) and AChE (PDB: **4EY6**) were carried out. The validation of the docking protocol and the choice of the crystal MAO-B and AChE structures are reported elsewhere [42,43]. The validation process was carried out by implementing redocking, cross-docking and ligand enrichment which demonstrated that PDB 2V5Z was the best MAO-B crystal structure out of the deposited X-rays.

### 2.11. Molecular Docking in MAO-B

Two of the most active MAO-B inhibitors—**vh0** and **vh1**—as well as one weak inhibitor—**vh4**—were employed in the docking simulations against MAO-B. The generated docking conformations for the three ligands were dissimilar; however, in all cases, the pyrrole ring was situated between the entrance and substrate cavities. The ChemPLP fitness scores for **vh0**, **vh1** and **vh4** were 104.65, 91.04 and 59.99, respectively, which provided a good correlation with the in vitro results. When Glide was applied as a docking program, no results were returned, which could be due to the different searching and scoring algorithms of the programs. In addition, the MM/GBSA free binding energy was calculated which demonstrated the following scores—75.00, −61.48 and −14.25 for **vh0**, **vh1** and **vh4**, respectively.

**Figure 5:**
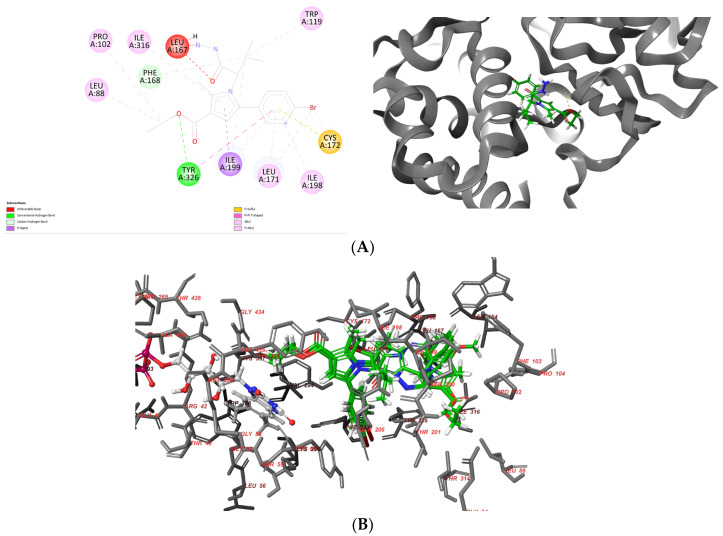
Interaction diagrams demonstrating the major intermolecular interactions of **vh0** (**A**) with the active site of MAO-B (PDB: **2V5Z**) and superimposing of the docked ligands—**vh0**, **vh1** and **vh4** (**B**).

**Vh0** formed one stable hydrogen bond with the active residue Tyr326 (Figure 5A). Moreover, numerous hydrophobic interactions with the active residues Leu88, Pro102, Trp119, Leu171, Ile198 and Ile316 were observed (Table 3).

Importantly, the pyrrole and benzene moieties in the **vh0** participated in several stabilizing forces with Leu171, Cys172, Ile198, Ile199 and Ile316, which demonstrates the significance of the aromatic structures for the binding MAO-B affinities. However, the acquired active conformation of the most active pyrrole derivative did not include the participation of any of the residues included in the “aromatic cage”, which are essential for the binding affinity [44]. In addition, one steric clash with Leu167 was detected. The former unfavorable bond was established due to the suboptimal conformation of **vh0** in the active site of MAO-B. Consequently, further modifications of the substituents in the pyrrole should be carried out to acquire more prominent ligands.

The active conformation of the second active pyrrole-based compound—**vh1**, demonstrated similar problems (Figure S16A). However, here the compound was situated deeper into the active gorge and the ethyl ester fragment was facing the ‘aromatic cage’. Therefore, Tyr398, Tyr435 and FAD participated in several weak hydrophobic interactions with **vh1**. Moreover, Tyr398 formed π–π interaction with the aromatic core of pyrrole. The distance of the former bond was 4.41 Å, which suggests its insignificant effect on the stability of the complex. An optimal conformation could be observed when an aromatic ring is in close vicinity to the residues participating in the formation of the “aromatic cage” [45]. A single hydrogen bond with the active amino residue Tyr188 was observed. Tyr326 participated in a steric clash with the p-bromophenyl residue.

The inactive compound **vh4** was also involved in the molecular docking simulations to analyze the detected low in vitro results (Figure S16B). The ChemPLP fitness score of the compound was calculated to be 59.99, which is significantly lower compared to **vh0**. Importantly, five unfavorable clashes were formed by the active residues Leu171, Ile198, Gln206, Met341 and Tyr326 which corresponds to the unavailability of **vh4** to fully accommodate the active site of MAO-B. Thus, further modifications of the structure are needed.

Overall, the molecular docking simulations demonstrated the inability of most of the novel valine-based ligands to fit properly in the active site of MAO-B, which should be considered in the subsequent design of potent pyrrole-based MAO-B inhibitors. The most steric clashes were detected between **vh4** (inactive MAO-B inhibitor) and the active site of the protein.

### 2.12. Molecular Docking in AChE

Previous findings demonstrated that PDB–4EY6 shows excellent results after redocking, cross-docking and ligand enrichment validation processes, thus the former was applied in the docking studies [43]. The GOLD 5.3 demonstrated that the best-suited title compound in the active site of AChE is **vh0** with ChemPLP score of 125.45. The second best scored ligand was **vh2** with a score of 105.08. The hydrazide–hydrazones **vh1**, **vh3** and **vh4** were given scores of 100.60, 100.71 and 91.83, respectively. In addition, the MM/GBSA free binding energies were calculated which showed the following scores—81.47, −57.42, −69.15 and −38.91 for **vh0**, **vh1**, **vh2** and **vh4**, respectively. Initially, Glide returned no docking poses; however, after increasing the Grid space using the Receptor Grid Generation module, good-to-moderate scores were observed (Table 4).

Good correlation of the observed consensus docking scores was detected. The utilization of MM/GBSA indicated **vh0** as the most active with a score higher than the standard co-crystallized ligand Galantamine. However, the former module did not provide a scoring of **vh3** which was experimentally tested as a weak AChE inhibitor. Therefore, the MM/GBSA recalculations should not be considered as most robust in the examined case. It could be noted that the most active in vitro AChE inhibitor **vh0** (62% at 10 μM concentration) provided the most stable conformation within the active site of AChE using in silico calculations. Importantly, the molecular docking studies correlated well with the in vitro data which is related to the good fitness score (125.45) and XP score (−9.78 kcal/mol) of **vh0**. However, the ligands **vh2** and **vh4**, which exerted no inhibition activity against the AChE enzyme, displayed moderate to good docking scores of 105.08 and 91.83, respectively. Moreover, moderate scores of **vh2** and **vh4** were obtained with the second docking program—Glide. The presented docking simulations in AChE confirms the main drawback of the in silico studies—the high risk of false-positive results.

**Figure 6:**
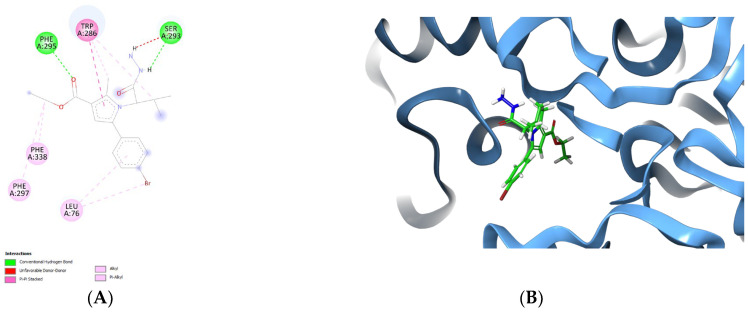
Active conformation of **vh0** in the active site of **4EY6** displayed in 2D (**A**) and 3D panels (**B**).

To obtain further details for the stabilizing forces, the active conformation of the best AChE inhibitor—**vh0**, was analyzed in the active site of the enzyme. The visualized complex **4EY6-vh0** in the active site of AChE is provided in Figure 6.

Notably, the observed intermolecular interactions with the active site of AChE were hydrophobic in nature. The former bonds were described in the stabilization of several active AChE inhibitors [38,46]. The active amino acids Leu76, Phe297 and Phe338 were involved in π−alkyl stabilizing bonds with fragments of the hydrazide **vh0**. The pyrrole fragment was situated in the substrate pocket, while the ethyl ester group was facing the solvent accessible site of the enzyme. A π–π stacking interaction was formed between the pyrrole core structure and the active amino acid Trp286, which highlights the positive role of the fragment for the formation of stable complex with AChE. Notably, two hydrogen bonds were formed. The hydrogen bond formation plays an important role in the formation of stable complexes with potent AChE inhibitors [47]. Ser293 participated in one hydrogen bond with the NH-group of the hydrazide moiety, as well as one steric clash with the second NH_2_ group. The active amino acid Phe295 was also involved in the formation of a H-bond with the carbonyl group from the ethyl ester group. Interestingly, the title hydrazide—**vh0**, was not situated in the peripheral site of the AChE—PAS, which decreases the final docking score. It could be hypothesized that the small size of **vh0** leads to low docking score and not compact localization in the active site of AChE (PDB: **4EY6**).

### 2.13. ADME Analysis

As a following stage, we carried out an in silico ADME analysis and BBB-PAMPA to determine the pharmacokinetic properties of **vh0** and **vh1**. For the former purpose, we employed the QikProp module in Maestro (Table 5).

Considering the obtained in silico data, compound **vh0** demonstrated the most promising ADME properties with potential 100% oral absorption. Moreover, the most active dual MAO-B/AChE inhibitor obeys the Rule of Five, which was not observed in the case of vh1. Two possible metabolites of vh0 could be formed. Importantly, the obtained brain/blood partition coefficients (QPlogBB) of **vh0** and **vh1** were optimal at −1.002 and −0.415, respectively. The given ranges of Qikprop were −3.0 to 1.2. The former physicochemical parameter is a necessity for the design of novel MAO-B and AChE inhibitors [48].

The simultaneous utilization of several servers for the evaluation of the ADME parameters is needed as reported by Dulsta et al. [49]. The authors noted that out of 18 analyzed free web servers, ADMETLab offers the best accuracy and precision. Thus, the former was utilized for the ADME calculations of the best dual-acting inhibitor—**vh0** (Table 6). The calculations demonstrated that **vh0** obeys the Rule of Five, the Pfizer Rule and the Golden Triangle. However, the GSK Rule was not met considering the molecular weight of the hit compound 422.22, which is slightly higher than the required 400. Moreover, the tested multi-target molecule showed a high probability of interacting and inhibiting CYP2C9 and CYP3A4 isoforms, which will be experimentally validated by in vitro tests in future work.

### 2.14. BBB-PAMPA Permeability Test

A good penetration through the blood–brain barrier is required for novel compounds targeting AD. Thus, the passive diffusion of **vh0** was evaluated using an in vitro permeability test. Values of effective permeability coefficient Pe, presented in terms of –logPe are provided in Table 7 for the evaluated compound.

The PAMPA evaluation demonstrated that the blood–brain barrier (BBB) permeability of the active valine-based dual inhibitor (**vh0**) is 5.451, which is in the medium range compared to the applied references. Theophylline, corticosterone, propranolol hydrochloride, and lidocaine were applied as references for low, medium and high permeability, respectively. The passive diffusion could not be influenced by the ionization of **vh0** because the compound is weak acid (pKa 10.57) and it exists as neutral molecules at physiological pH 7.4. Overall, the experimental result provided good correlation with the in silico studies.

### 2.15. DFT Studies

#### Structural and Molecular Electrostatic Potential Analysis

The structure of compounds **vh0** and **vh1** were optimized at B3LYP/6-311G^++^(d,p). The changes in the bond lengths and angles of **vh0** were presnted to illustrate the effect of optimization. Together with this, C25-C24, C22-C21, C21-C6, C15-C14, and C4-C3 bond lengths were longer after optimization. On the other hand, most of the heteroatom-consisting bonds had a higher bond length after optimization. Moreover, some of them provided a shorter bond length with optimization (Supplementary Table S1). Similarly, changes in bond angles were detected with the optimization. An experimental X-ray analysis of the Br-C-C bond angle revealed a value of 119.1°. Similarly, the bond angle from an earlier DFT analysis was found to be 119.0811° and 119.1483° for the Br-C-C connection [52]. In this study, the Br26-C23-C24 and Br26-C23-C22 had a bond angle of 119.4930° and 119.5901°, respectively. These values were near the theoretical and experimental bond angles reported earlier. The C-C-C-containing bonds gave a lower bond angle after optimization but some of them had a higher bond angle value. In general, the heteroatom-consisting bond angles had lower values after optimization. Some of such types of bonds had a higher bond angle with the optimization (Supplementary Table S1). Previous studies reported a bond angle of 118.1°–119.163° for C–O–C bonds [53,54]. In this study, the optimized structure had a bond angle of 119.8903° for C7–O9–C10 after optimization. This result was in line with the reported studies. Similarly, various studies reported a range of bond angles from 115.7° to 124.8° for C–C–O bonds [53,54]. In this study, the optimized structure had a bond angle of 122.9431°, 125.7615°, and 112.6416° for C13–C17–O18, C3–C7–O8, and C3–C7–O9, respectively. The values obtained from this study were similar to the previous findings with a slim difference. The bond angle for N1–C2–C12 was found to be 112.1° in one study and 113.4° in another study [54,55]. In this study, the bond angles for N1–C13–C17 and N1–C13–C14 were found to be 111.9018° and 113.4433°, respectively. These values were compatible with the previous studies. Bond angles with values ranging from 105.8° to 124.5° were reported for C-N-C [56]. Similarly, the C-N-C bond angles in this study were in the 108.7989°–128.1032° range (Supplementary Table S1). Furthermore, the bond lengths were assessed in light of the literature. The values obtained were within an acceptable range [54].

**Figure 7:**
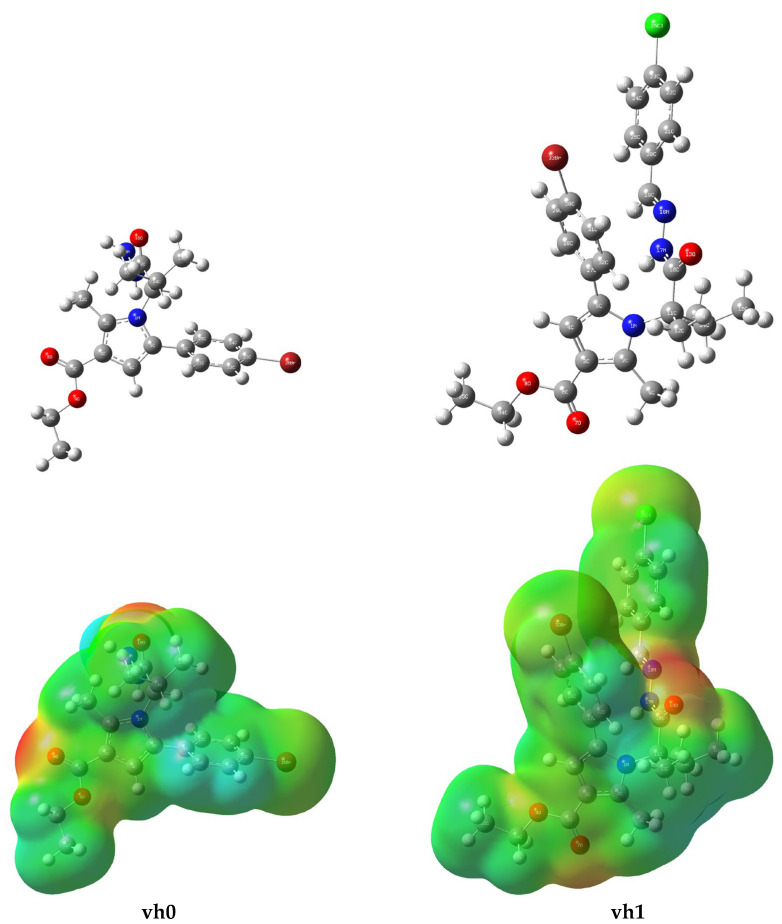
Optimized structure (**upper panel**) and MEP (**lower panel**) of compounds **vh0** and **vh1**.

The molecular electrostatic potential (MEP) maps of **vh0** and **vh1** were drawn from the DFT results to obtain an insight into the distribution of electrophilic and nucleophilic regions on the compounds. The electrostatic landscapes of the compounds were visualized and classified as predominantly red (negative) and blue (positive) regions. The predominantly blue regions are nucleophilic reactivity parts of a molecule whereas the predominantly red regions are electrophilic reactivity parts. The electrophilic reactivity of **vh0** and **vh1** might arise mainly from the vicinity of the oxygen atoms in their structure (Figure 7). In this regard, the oxygen atoms in the carboxylate functional group and the oxo of the butane are expected to contribute much to this. In addition, the nitrogen of the hydrazine group of **vh1** might contribute to this reactivity to some extent. Similarly, the nucleophilic reactivity might arise from the hydrogen atoms of **vh0** and **vh1**, especially from the hydrogens of the bromophenyl, ethyl, and butane (Figure 7).

### 2.16. FMO Analysis

From the DFT energy computations, FMO analysis, especially, the highest occupied molecular orbital (HOMO) and the lowest unoccupied molecular orbital (LUMO) energies of **vh0** and **vh1** were obtained (Table 8). From HOMO and LUMO energies, the following parameters were computed: Ionization potential (IP = −E_HOMO_), electron affinity (A = −E_LUMO_), hardness (η = (I − A)/2), softness (S = 1/2η), Mulliken electronegativity (ᵡ = (I + A)/2) [57], electrophilicity index (ꞷ = µ2/2η) [58], chemical potential (µ = -(I + A)/2), maximum charge transfer (ΔNmax = (I + A)/2(I − A)) [59].

The HOMO and LUMO energies are important as they give a general notion about the electronic exchange capacity of a compound. The HOMO energy of compound **vh0** was found to be in the standard range but slightly lower than the value of **vh1**. On the other hand, the LUMO energy was found to be high relative to **vh1** and the reported small molecules with similar complexity [54,60,61,62,63]. The LUMO energy is the reflection of the compound’s capacity for accepting electrons. Therefore, **vh0** is anticipated to have high potential for accepting electrons relative to **vh1**. Similarly, the energy gap (∆E) between the LUMO and HOMO energies of the **vh0** was high relative to **vh1** and the reported values [54,60,61,62,63]. The higher the energy gap of a compound, the higher its chemical stability. Hence, compound **vh0** is expected to have high chemical stability relative to **vh1** (Table 8).

**Figure 8:**
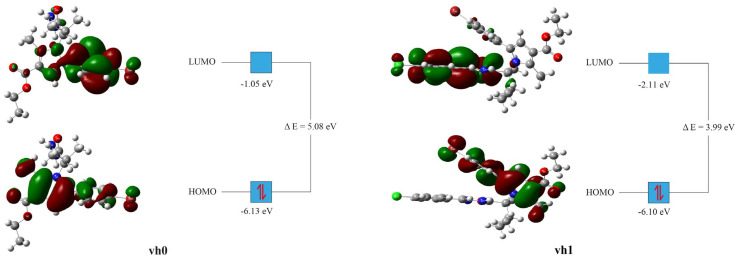
Frontier molecular orbitals of **vh0** and **vh1** at B3LYP/6-311G^++^(d,p) state.

The HOMO and LUMO orbital distributions for compounds **vh0** and **vh1** were somewhat different (Figure 8). The HOMO orbitals of the two compounds were distributed similarly. The HOMO orbitals were concentrated mainly around the pyrrole heterocyclic ring and the bromophenyl ring. The methyl substituent of the pyrrole and the ethyl substituent of the carboxylate group were also surrounded by the HOMO orbitals. The only discern of **vh1** was the absence of HOMO orbitals on the ethyl of its carboxylate group. On the other hand, the LUMO orbitals of the two compounds are distributed differently. The LUMO orbitals of **vh0** were mainly concentrated around the bromophenyl ring. Sparse LUMO orbitals were observed around the pyrrole heterocyclic ring, the carboxylate group, and the oxo on the butane. The bromophenyl of **vh0** was found to be in a high concentration vicinity for both HOMO and LUMO orbitals (Figure 8). Therefore, this part is expected to facilitate the electron exchanges of the compound. The LUMO orbitals of **vh1** were mainly concentrated around the 4-chlorobenzylidene hydrazinyl part. The LUMO orbitals on the bromophenyl heterocyclic ring were sparse.

## 3. Materials and Methods

Reactants and solvents were obtained from commercial suppliers (Sigma Aldrih and Acros, Merck KGaA, Darmstadt, Germany), and used without further purification. The microwave-assisted syntheses were performed in a FlexiWave Milestone Lab reactor, Sorisole, Italy (equipped with a fibre optic and IR sensors). For the monitoring of the reactions thin layer chromatography (TLC) was applied. The TLC characteristics were measured on Kieselgel 60, F254 (Darmstadt, Germany) at ambient temperature, employing a mobile phase: chloroform:ethanol (10:0.6). The melting points were determined by Kruss M5000 (Hamburg, Gemrany) and were uncorrected. The absorbance was measured in a multi-plate reader Synergy 2 (BioTek Instruments, Haverhill, MA, USA). The IR spectra range was registered on a Nicolet iS10 FT-IR spectrometer, Smart iTR adapter (Thermo Fisher Scientific, Waltham, MA, USA). The 1H spectra were registered on Bruker Avance Neo 400 (Biospin GmbH, Rheinstetten, Germany) and 100 MHz for 13C. The mass spectrums were measured on a 6410 Agilent LCMS triple quadrupole mass spectrometer (LCMS) with an electrospray ionization (ESI) interface (Agilent Technologies, Lexington, MA, USA).

### 3.1. Chemistry

1,4-Dicarbonyl compounds (**2**): The synthesis of the dicarbonyl compound (**2**) was accomplished according to a procedure reported by A. Bijev [64].

N-pyrrolylcarboxylic acid (**3**): 0.1 mol ethyl 2-acetyl-4-(4-bromophenyl)-4-oxobutanoate (**2**) and 0.12 eq. of the amino acid valine were dissolved in 15 mL of glacial acetic acid. The mixture was heated by a microwave reactor for 40 min. The synthetic conditions were optimized by applying MW irradiation (FlexiWave Milestone Lab reactor) which reduced the reaction time of the obtained valine-based acid [65].

Ethyl ester of the N-pyrrolylcarboxylic acid (**4**): An esterification with thionly chloride and ethanol was carried out. SOCl_2_ (0.1 mol) was included dropwise to the N-pyrrolylcarboxylic acid (**3**) (0.05 mol) diluted in cold abs. ethanol. After 30 min the mixture was placed in MW reactor which was set at 75 °C and 750 W for 40 min. Subsequently, the solvent was eliminated using rotary evaporator and the oil was washed with Na_2_CO_3_. The final product was incorporated in the next synthetic phase without isolation.

Hydrazide of the N-pyrrolylcarboxylic acid (**vh0**): The hydrazinolysis was carried out by reacting 0.04 mol of the ethyl ester of the N-pyrrolylcarboxylic acid (**4**) and 0.16 mol hydrazine hydrate in 30 mL of abs. ethanol. The heating was performed in conventional conditions and in a MW reactor. The reaction was completed in 96 hr after conventional heating and in 1 hr (750 W) after applying MW irradiation. After the formation of the crystals, the hydrazide was filtered and washed with cold abs. ethanol. The recrystallization was conducted with ethanol.

Ethyl-5-(4-bromophenyl)-1-(1-hydrazineyl-2-isopropyl-1-oxopropan-2-yl)-2-methyl-1H-pyrrole-3-carboxylate (**vh0**): The valine-based hydrazide was obtained according to the aforementioned general procedure. Yield 87%; Melting point 121.2–121.8 °C; Rf 0.35 (10:0.4); purity > 95% (HPLC); IR vmax: 3335 (NH_2_), 2963 (NH), 1670 (C=O), 774 (p-disubstituted C_6_H_4_), 587 (Br); ^1^H NMR (DMSO, 400 MHz) δ 9.26 (s, 1H, NH_2_), 8.90 (2H, s, H-2, H-6 (bromobenzene)), 7.64 (2H, d, J = 8.40 Hz, H-3, H-5), 7.45 (1H, s, (4-CH)), 6.45 (2H, s, NH-NH_2_), 4.45 (2H, m, CH_2_-CH_3_), 4.15 (1H, d, J = 10.70 Hz, CH-CONH), 3.35 (3H, s, CH_3_), 2.64 (1H, m, CH-(CH_3_)_2_), 2.45 (3H, t, J = 7.10 Hz, CH_2_-CH_3_), 0.85 (3H, d, J = 6.40 Hz, CH_3_ (isopropyl)), 0.45 (3H, d, J = 6.90 Hz, CH_3_ (isopropyl)); ^13^C NMR (100 MHz) δ ppm: 11.8, 14.1, 18.8, 27.4, 60.9, 76.1, 104.7, 108.2, 123.1, 128.3, 132.1, 132.7, 165.9, 174.1; *m*/*z* (FTMS + pESI) 423.10 [M + 2H^+^].

Schiff bases (**vh1**-**vh4**): The hydrazide–hydrazones bases were synthesized using reaction of vh0 (hydrazizde) with different aldehydes (1–4) by both conventional heating and microwave radiation. Glacial acetic acid was applied as a catalyst and a solvent. The conventional conditions led to reaction times of 30–50 min. The microwave radiation lowered the reaction times to 1–3 min. with increased yields. The completed reactions (detected by TLC) were poured into cold water and the crystals were filtered. The hydrazide–hydrazones were washed with hexane and recrystallized from ethanol/water.

Ethyl-5-(4-bromophenyl)-1-(1-(2-(4-chlorobenzylidene)-hydrazineyl)-2-isopropyl-1-oxopropan-2-yl)-2-methyl-1H-pyrrole-3-carboxylate (**vh1**): Yield 76%; Melting point 157.5–158.1 °C; Rf 0.60 (10:0.4); purity >95% (HPLC); IR vmax: 2966 (NH), 1674 (C=O), 1570 (C=N), 820 (p-disubstituted C_6_H_4_), 763 (Cl), 559 (Br); ^1^H NMR (DMSO, 400 MHz) δ 11.64 (s, 1H, NH), 11.22 (1H, s, N=CH), 7.90 (1H, s, (4-CH)), 7.82 (2H, t, J = 7.40 Hz, H-2′, H-6′ (chlorobenzene)), 7.57 (2H, d, J = 8.10 Hz, H-2, H-6 (bromobenzene)), 7.50 (2H, d, J = 8.10 Hz, H-3, H-5), 2.86 (2H, t, J = 7.25 Hz, H-3′, H-5′), 2.64 (2H, q, J = 7.50 Hz, CH_2_-CH_3_), 2.55 (1H, d, J = 9.20 Hz, CH-CONH), 1.93 (3H, s, 2-CH_3_), 0.90 (1H, m, CH-(CH_3_)_2_), 0.85 (3H, d, J = 6.60 Hz,CH_3_ (isopropyl)), 0.45(3H, d, J = 6.70 Hz,CH_3_ (isopropyl)); ^13^C-NMR (100 MHz) δ ppm: 11.8, 14.1, 18.8, 27.4, 31.9, 60.9, 76.4, 104.7, 108.2, 116.0, 123.1, 128.3, 129.3, 132.1, 135.5, 139.0, 142.1, 154.7, 165.9, 174.8; *m*/*z* (FTMS + pESI) 545.09 [M + 2H^+^].

Ethyl-5-(4-bromophenyl)-1-(1-(2-(3-fluorobenzylidene)-hydrazineyl)-2-isopropyl-1-oxopropan-2-yl)-2-methyl-1H-pyrrole-3-carboxylate (**vh2**): Yield 80%; Melting point 151.2–151.9 °C; Rf 0.65 (10:0.4); purity > 95% (HPLC); IR vmax: 2967 (NH), 1679 (C=O), 1579 (C=N), 830 (p-disubstituted C_6_H_4_), 781 (F), 592 (Br); ^1^H NMR (DMSO, 400 MHz) δ 11.72 (s, 1H, NH), 11.55 (1H, s, N=CH), 8.41 (1H, s, H-2′ (fluorobenzene)), 7.90 (1H, s, (4-CH)), 7.73 (1H, t, J = 6.50 Hz, H5′), 7.52 (2H, q, J = 8.40 Hz, CH_2_-CH_3_), 7.48 (2H, d, J = 8.10 Hz, H-2, H-6 (bromobenzene)), 7.41 (1H, d, J = 7.20 Hz, H-6′), 7.32 (2H, d, J = 8.10 Hz, H-3, H-5), 7.12 (1H, d, J = 7.40 Hz, H-4′), 7.05 (1H, d, J = 10.50 Hz, CH-CONH), 2.51 (3H, s, 2-CH_3_), 0.90 (1H, m, CH-(CH_3_)_2_), 0.74 (3H, d, J = 6.40 Hz,CH_3_ (isopropyl)), 0.53 (3H, d, J = 6.90 Hz,CH3 (isopropyl)); ^13^C-NMR (100 MHz) δ ppm: 11.8, 14.1, 18.8, 27.4, 31.9, 60.9, 76.4, 104.7, 108.2, 123.1, 128.3, 129.3, 130.2, 132.1, 135.5, 139.0, 142.1, 154.7, 162.8, 174.8; *m*/*z* (FTMS + pESI) 529.12 [M + 2H^+^].

Ethyl-5-(4-bromophenyl)-1-(1-(2-(4-methoxybenzylidene)-hydrazineyl)-2-isopropyl-1-oxopropan-2-yl)-2-methyl-1H-pyrrole-3-carboxylate (**vh3**): Yield 63%; Melting point 157.3–158.1 °C; Rf 0.75 (10:0.4); purity > 95% (HPLC); IR vmax: 2966 (NH), 1668 (C=O), 1572 (C=N), 830 (p-disubstituted C_6_H_4_), 595 (Br); ^1^H NMR (DMSO, 400 MHz) δ 11.48 (s, 1H, NH), 11.31 (1H, s, N=CH), 7.90 (1H, s, (4-CH)), 7.64 (2H, t, J = 7.40 Hz, H-2′, H-6′ (methoxybenzene)), 7.52 (2H, d, J = 8.10 Hz, H-2, H-6 (bromobenzene)), 7.13 (2H, d, J = 8.10 Hz, H-3, H-5), 6.95 (2H, q, J = 7.50 Hz, CH_2_-CH_3_), 3.85 (2H, t, J = 7.25 Hz, H-3′, H-5′), 2.84 (1H, d, J = 8.30 Hz, CH-CONH), 2.55 (3H, s, O-CH_3_), 1.92 (3H, s, 2-CH_3_), 0.90 (1H, m, CH-(CH_3_)_2_), 0.77 (3H, d, J = 7.20 Hz, CH_3_ (isopropyl)), 0.49 (3H, d, J = 6.40 Hz,CH_3_ (isopropyl)); ^13^C-NMR (100 MHz) δ ppm: 11.8, 14.1, 18.8, 27.4, 31.9, 55.8, 60.9, 76.4, 104.7, 108.2, 114.2, 123.1, 129.7, 130.0, 132.1, 135.5, 139.0, 142.1, 157.6, 165.9, 174.8; m/z (FTMS + pESI) 541.14 [M + 2H^+^].

Ethyl-5-(4-bromophenyl)-1-(1-(2-(2,4-dimethoxybenzylidene)-hydrazineyl)-2-isopropyl-1-oxopropan-2-yl)-2-methyl-1H-pyrrole-3-carboxylate (**vh4**): Yield 55%; Melting point 147.4–148 °C; Rf 0.80 (10:0.4); purity >95% (HPLC); IR vmax: 2966 (NH), 1673 (C=O), 1558 (C=N), 830 (p-disubstituted C6H4), 569 (Br); ^1^H NMR (DMSO, 400 MHz) δ 11.65 (s, 1H, NH), 11.14 (1H, s, N=CH), 7.92 (1H, s, (4-CH)), 7.72 (2H, d, J = 9.20 Hz, H-3, H-5), 7.56 (2H, d, J = 8.30 Hz, H-2, H-6 (bromobenzene)), 7.50 (2H, q, J = 8.30 Hz, CH_2_-CH_3_), 7.42 (1H, d, J = 6.50 Hz, H-6′ (methoxybenzene)), 6.51 (2H, d, J = 6.80 Hz, H-3′, H-5′), 4.42 (1H, d, J = 7.25 Hz, CH-CONH), 2.84 (3H, 4-O-CH_3_), 2.68 (3H, 2-O-CH_3_), 2.35 (3H, s, 2-CH_3_), 0.97 (1H, m, CH-(CH_3_)_2_), 0.77 (3H, d, J = 7.10 Hz,CH_3_ (isopropyl)), 0.50 (3H, d, J = 7.10 Hz,CH_3_ (isopropyl)); ^13^C-NMR (100 MHz) δ ppm: 11.8, 14.1, 18.8, 26.0, 27.4, 31.9, 55.8, 56.1, 60.9, 76.4, 100.3, 104.0, 108.2, 114.2, 120.1, 129.7, 131.0, 132.1, 135.5, 139.0, 142.1, 154.7, 158.6, 165.9, 174.8; m/z (FTMS + pESI) 571.15 [M + 2H^+^].

### 3.2. DPPH Assay

The radical scavenging activities of the title compounds were examined with a DPPH (2,2- diphenyl-1-picrylhydrazyl) test. A solution of DPPH has an intense violet color and a UV absorption at 515 nm could be observed. The presence of an antioxidant leads to visible decolorization of the sample and the absorption could be measured. Here, the scavenging rate of the DPPH radical was carried out by the widely employed classical protocol of Brand-Williams et al. [66]. A 1 mg/mL of the title compounds were reacted with methonol solution of the DPPH (1 mmol/L). Thereafter, the obtained mixture was left in a dark palce for 15 min. The absorbance was assessed at 517 nm. Three measurements were conducted for each sample and Trolox was used as a standard. The percentage inhibition of the tested samples was calculated by the following Formula (1):

DPPHscavenging activity = (Abscontrol − Abssample)*/*Abscontrol × 100% (1)

where Abscontrol is the absorbance of the DPPH radical in methanol and Abssample is the absorbance of the DPPH radical solution mixed with the sample.

### 3.3. ABTS Assay

The radical scavenging properties of the newly synthesized structures were also calculated with the help of a ABTS test according to a modified method of Arnao et al. [67]. The tests were conducted in methanol at room temperature. The absorbance of the solutions was measured at λ = 734 nm. The stable ABTS radical was produced by mixing 7 mmol/L solution of ABTS and 2.4 mmol/L solution of potassium persulphate, which were allowed to react for 14 h in the dark at room temperature. The working solutions were made of 2 mL of the stock solution diluted in 50 mL of methanol with an absorbance of 0.30 ± 0.04 units at 517 nm. In total, 1 mL of the ABTS working solution was allowed to react with the pyrrole derivatives with concentrations of 1 mg/mL for 10 min. The inhibition percentage was calculated by applying the same formula as the DPPH assay.

### 3.4. hMAOB Enzyme Assay

The examined compounds were evaluated for possible inhibitory capacity on human recombinant MAOA and MAOB enzymes by using the Amplex UltraRed (Sigma Aldrih and Acros, Merck KGaA, Darmstadt, Germany) reagent with some modifications [65]. All reagents were prepared in accordance with the producer’s instructions. As a substrate the tyramine hydrochloride was used for *h*MAO-B and p-benzylamine for *h*MAO-A. The reaction mixture included a stock solution of Amplex Red, the reaction buffer at pH 7.4 and the storage solution of horseradish peroxidase (10 U/mL). Five concentrations of the exam compounds were applied. The combination: compounds + *h*MAOA/B was placed in a 96-well plate, and then the plate was incubated for 30 min (dark, at 37 °C). The reaction was started by the addition of 50 μL of the mix solution: Amplex Red, horseradish peroxidase, and tyramine/p-benzylamine, as an enzyme substrate, in the reaction buffer. The fluorescence was measured every 30 min until 150 min, in dark, while shaking the reaction mixture at constant temperature of 37 °C. The fluorimetric measurements were assessed using a Synergy 2 Microplate Reader (BioTek, Winooski, VT, USA).

### 3.5. In Vitro AChE Assay

The AChE potentials of the new valine-based molecules were evaluated according to a modified Ellman’s method [68]. Initially, stock solutions (1 mg/mL) of the new structures were diluted in DMSO and five solutions were prepared by dilutions in order to access the inhibitory potential in different concentrations. Thereafter, the pyrrole-based molecules were incubated with sodium phosphate buffer, and AChE solution for ten minutes at 37 °C. The reaction was started by the addition of 5,5-dithio-bis-(2-nitrobenzoic acid) (DTNB) (10 mM; 20 μL) and acetylthiocholine iodide (14 mM; 20 μL). The absorbance was measured at 412 nm by a microplate reader against a blank DMSO. The percent inhibition activity was measured against the blank probe. Donepezil was utilized as a standart acetylcholinesterase inhibitor. The results were expressed as a mean ± SD (n = 3). Values of *p* < 0.05 were observed as statistically significant.

### 3.6. Statistical Analysis

The in vitro results (MAO-A, MAO-B and AChE inhibitory capacities) were expressed as a mean ± SD (n = 3). Values of *p* < 0.05 were considered statistically significant. Compounds’ concentrations providing 50% inhibition (IC_50_) were obtained by plotting the percentage inhibitions against compound concentrations.

### 3.7. Molecular Docking

The docking simulations were performed with GOLD 5.3 and Glide (Maestro). The crystal structures of MAO-B co-crystallized with safinamide (PDB code: **2V5Z**) [69] and acetylcholinesterase co-crystallized with galantamine (PDB code: **4EY6**) [70] were retrieved from the Protein Data Bank (PDB) (https://www.rcsb.org accessed on 15 May 2023). The validation of the crystal structures is reported elsewhere [42,43].

The preparation of the crystal structures was accomplished with the protein preparation module in Schrödinger. The active sites of the enzymes were created around the active conformations of the corresponding co-crystallized ligands. The title compounds were drawn with the 2DSketcher (Maestro, Schrödinger Release 2024-1: Maestro, Schrödinger, LLC, New York, NY, USA, 2024. and converted to their corresponding 3D structures with the LigPrep module in Maestro. Moreover, hydrogens were added, bond order was assigned and the title structures were minimized by a OPLS4 force field. Recalculations of the binding free energies of the newly formed complexes was calculated with the Molecular Mechanics/Generalized Born Surface Area (MM-GBSA) of Prime (Maestro).

### 3.8. In Silico ADME

To analyse the physicochemical features of the novel valine-based compounds, in silico ADME calculations were carried out with the QikProp module in Schrodinger (Schrödinger Release 2024-1: QikProp, Schrödinger, LLC, New York, NY, USA, 2024). The theoretical in silico simulations examine the properties of the novel compounds and compares them with 95% of the known drugs [48].

### 3.9. PAMPA-BBB Assay

The in vitro Parallel Artificial Permeability Assay—blood brain barrier (PAMPA-BBB) test was carried out by PAMPA Permeability Analyzer (pIONInc, Billerica, MA, USA). The permeabilities (Pe) of the dual-acting pyrrole-based hydrazide—**vh0**, were evaluated considering the manufacturer BBB Protocol. Initially, the stock solutions of **vh0** and reference molecules were dissolved in DMSO and diluted with Prisma HT buffer pH 7.4 (Pion Inc. Billerica, MA, USA) to reach a concentration of 50 μM. The well plates were filled with 200 µL BSB (Brain Skin Buffer). The incubation was established for 4 h at 25 °C with no stirring. The permeability of the structures was evaluated by accessing their concentrations in both the donor and acceptor phases spectrophotometrically. The permeability coefficients were calculated by PAMPA Explorer Command Software and presented as –logPe. A structure is classified as highly permeable if –logPe < 5 and low permeable if –logPe > 6. When 6 < –logPe < 5 the molecule is identified with a medium permeability [71]. Theophylline, corticosterone, lidocaine and propranolol hydrochloride were applied as references for low, medium and high permeability, respectively [50,72].

### 3.10. DFT Studies

A DFT study of the most active compounds, **vh0** and **vh1**, was performed using Gaussian 09. The structure of compounds **vh0** and **vh1** were optimized and then energy was computed using the same setup. The method setup was DFT/B3LYP with a basis set of 6-311G at the ground state. Thereafter, structural, electrostatic potential and frontier molecular orbital (FMO) analyses were performed after the necessary parameters were calculated using the related formulas. Visualization of the calculation results was performed using Gaussview 5.0. The DFT study was performed according to a reported study [73].

## 4. Conclusions

We designed and synthesized a series of pyrrole-based ligands as multifunctional drugs in the treatment of AD. Among them, **vh0** was selected as a potential lead compound for further development due to its selective MAO-B (IC_50_ *h*MAOB—0.665 μM; SI >150) and AChE (IC_50_ *ee*AChE—4.145 μM) inhibitory activities, moderate radical scavenging properties, as well as its good experimental blood–brain permeability.

The molecular docking studies provided the active conformation of **vh0** in the active sites of MAO-B and AChE. Interestingly, several functional groups of **vh0** were involved in steric interactions with active amino acids and further lead optimizations were suggested. The frontier molecular orbitals and the electrochemical properties of **vh0** and **vh1** were calculated using DFT studies at a 6-311G basis set in the ground state. These findings indicate that **vh0** is a feasible hit structure in the search for novel multi-target-based compounds for the treatment of AD. Further in vivo studies are planned to validate the obtained experimental data and investigate the toxicological profile of the hit molecule **vh0**.

## Figures and Tables

**Table 1:** Comparison of the reaction times and yields of conventional and MW synthesis of pyrrole-based compounds.

Compound	Reaction Time		Yield (%)	
	Microwave Synthesis	Conventional Synthesis	Microwave Synthesis	Conventional Synthesis
**vh0**	1 h	96 h	87%	85%
**vh1**	1 min	30 min	92%	76%
**vh2**	1 min	40 min	91%	80%
**vh3**	3 min	50 min	94%	63%
**vh4**	3 min	50 min	88%	55%

**Table 2:** IC_50_ (EC_50_) of compounds **vh0-vh4**, and reference compounds on human monoamine oxidase (*h*MAO A/B) and acetylcholinesterase (AChE).

Compounds	IC_50_ (EC_50_), (μM± SD) ^a^ *h*MAOB	IC_50_ (EC_50_), (μM ± SD) ^a^ *h*MAOA	SI ^b^	IC_50_ (EC_50_), (μM ± SD) ^a^ AChE
**vh0**	0.665 ± 0.20	>100	>150	4.145 ± 1.31
**vh1**	0.744 ± 0.18	>100	>134	18.834 ± 0.91
**vh2**	>100	>100	>1	>100
**vh3**	>100	>100	>1	16.262 ± 2.15
**vh4**	>100	>100	>1	>100
Selegiline	0.330 ± 0.09	-	-	-
Chlorgyline	-	17.72 ± 0.096	-	-
Donepezil	-	-	-	0.02 ± 0.17

^a^ Values are expressed as the mean standard error of the mean of at least three different experiments in quadruplicate. ^b^ SI: *h*MAOB selectivity index = IC_50_(*h*MAO-A)/IC_50_(*h*MAO-B).

**Table 3:** Docking scores and active monoamine oxidase B amino residues participating in the interactions with the newly synthesized compounds and Safinamide.

Molecule	Docking Scores	MM/GBSA kcal/mol	Hydrophobic Interaction	Pi-Sulfur	Hydrogen Bond	π–π Interaction	Steric Clashes
**vh0**	104.65	−75.00	Leu88, Pro102, Trp119, Phe168, Leu171, Ile198, Ile199, Ile316, Tyr326	Cys172	Tyr326	Ile199	Leu167
**vh1**	91.04	−61.48	Pro102, Trp119, Leu164, Leu167, Phe168, Leu171, Ile198, Ile199, Leu328, Tyr435, FAD1502	Cys172	Tyr188	Phe343, Tyr398	Tyr326 (several clashes with the p-bromophenyl ring)
**vh4**	59.99	−14.25	Leu88, Pro102, Phe103, Pro104, Trp119, Leu164, Leu167, Phe168, Leu171, Ile198, Ile199, Leu328, Tyr398, Tyr435	-	Cys172	Ile199	Leu171, Ile198, Gln206, Tyr326, Met341
Safinamide	144.51	−89.82	Tyr60, Phe103, Leu171, Ile199, Gln206, Phe343, Tyr398, Tyr435	Gln206	Ile199, FAD	Leu171, Ile199, Ile316, Tyr326	-

**Table 4:** Docking scores and AChE amino residues participating in the interactions with the newly synthesized compounds.

Compound	Docking Scores GOLD 5.3	Docking Scores Glide (XP Docking)	MM/GBSA (kcal/mol)	Hydrophobic Interaction	Hydrogen Bond	π–π Interaction
**vh0**	125.45	−9.78 kcal/mol	−81.47	Leu76, Trp 286, Phe297, Phe338, Tyr341	Phe295, Ser293	Trp286
**vh1**	100.60	−8.36 kcal/mol	−57.42	Leu76, Trp 286, Phe297, Phe295	Phe295	Trp286
**vh2**	105.08	−7.46 kcal/mol	−69.15	Leu76, Trp 286, Phe297, Phe295	Tyr341	Trp286
**vh3**	100.71	−9.15 kcal/mol	n/a	n/a	n/a	n/a
**vh4**	91.83	−8.02 kcal/mol	−38.91	Leu76, Tyr124, Phe297, Tyr337	Ser293	-
Galantamine	109.72	−8.84 kcal/mol	−79.82	Leu76, Tyr124, Phe295, Phe297, Tyr337, Phe338	Phe338	-

**Table 5:** ADME profile of the most promising multi-target inhibitors.

Compound	^(a)^ MW	^(b)^ Donor HB	^(c)^ Accept HB	^(d)^ QPLog Po/w	^(e)^ QPLog S	^(f)^ QPLog BB	^(g)^ Percent Human Oral Absorption	^(h)^ Rule of Five	^(i)^ Metab
**vh0**	422.22	3	5	3.894	−5.724	−1.002	100%	0	2
**vh1**	544.88	1	4.5	6.885	−7.752	−0.415	100%	2	2

^(a)^ Molecular weight (Range: 130.0–725.0); ^(b)^ hydrogen bond acceptors (Range: 2.0–20.0); ^(c)^ hydrogen bond donors (Range: 0.0–6.0); ^(d)^ octanol/water partition coefficient (Range: −2.0–6.5); ^(e)^ QplogS: Predicted aqueous solubility (Range −6.5–−0.5); ^(f)^ QPlogBB:Predicted brain/blood partition coefficient (Range from 3.0 to 1.2); ^(g)^ percent human oral absorption; ^(h)^ number of violations of Lipinski’s rule of five (Range: maximum is 4); ^(i)^ number of likely metabolic reaction (Metab).

**Table 6:** ADME properties of vh0 calculated by ADMETLab 2.0.

Compound	Rule of Five	^a^ Pfizer Rule	^b^ GSK Rule	^c^ GoldenTriangle	CYP2C9 Inhibitor	CYP3A4 Inhibitor
**vh0**	+	+	-	+	+++	+++

^a^ Pfizer Rule logP > 3; TPSA < 75. ^b^ GSK Rule MW ≤ 400; logP ≤ 4. ^c^ GoldenTriangle 200 ≤ MW > 500; −2 ≤ logD ≤ 5.

**Table 7:** pKa, LogP and −logPe for the most active dual MAO-B/AChE inhibitor vh0.

PAMPA ID	Mw g/mol	pKa ^1^	Consensus LogP SwissADME ^2^	PAMPA BBB −logPe	PAMPA BBB −logPe Literature [50]
**vh0**	422	10.57	3.95	5.451 ± 0.005	-
Theophylline	180	8.81	−0.11	6.960 ± 0.004	6.3
Corticosterone	346	13.86	2.41	5.282 ± 0.007	4.5
Propranolol HCl	296	9.53	2.56	4.949 ± 0.003	3.7
Lidocaine	234	7.7	2.50	4.625 ± 0.006	4.3

^1^ ACD Labs, v.9.08. ^2^ SwissADME [51].

**Table 8:** Electrochemical properties of compound **vh0** and **vh1** from the DFT study.

Parameters	*E*_total_ (Hartee)	*E*_HOMO_ (eV)	*E*_LUMO_ (eV)	Δ*E* (eV)	*IP* (eV)	*A* (eV)	*µ* (eV)	*η* (eV)	*ᵡ* (eV)	*S* (eV)	*ꞷ* (eV)	Δ*N*_max_
**vh0**	−31,053.1	−6.13	−1.05	5.08	6.13	1.05	−3.59	2.54	3.59	0.20	2.58	0.71
**vh1**	−121,646.3	−6.10	−2.11	3.99	6.10	2.11	−4.11	2.00	4.11	0.25	4.22	1.03

## Data Availability

The data presented in this study are available on request from the corresponding author.

## Supplementary Materials

The following supporting information can be downloaded at: https://www.mdpi.com/article/10.3390/ph17091171/s1, ^1^H NMR (Figures S1–S5), IR (Figures S6–S10) and HPLC chromatograms (Figures S11–S15) of the hydrazide **vh0** and the Schiff bases **vh1-4**. Molecular docking 2D and 3D interaction figures of the title compounds (Figure S16). DFT calculated bond lengths and bond angles of **vh0** is provided in Supplementary Table S1.
