# Supplementary material for: Novel Pyrrole Derivatives as Multi-Target Agents for the Treatment of Alzheimer’s Disease: Microwave-Assisted Synthesis, In Silico Studies and Biological Evaluation
Source: Pharmaceuticals (Basel). 2024 Sep 4;17(9):1171. doi: 10.3390/ph17091171 (PMC11435393; doi:10.3390/ph17091171)
Supplement: Supplementary file 1 [file pharmaceuticals-17-01171-s001.zip › pharmaceuticals-3089735-supplementary.pdf]

Novel pyrrole derivatives as multi-target agents for the treat-ment of Alzheimer's disease: Microwave-assisted synthesis, in silico studies and biological evaluation

Emilio Mateev*[1], Valentin Karatchobanov[1], Marjano Dedja[1], Konstantinos Diamantakos[1], Alexandrina Mateeva[1], Muhammed Tilahun Muhammed[2], Ali Irfan[3], Magdalena Kondeva-Burdina[4], Iva Valkova[5], Maya Georgieva[1], Alexander Zlatkov[1]

[1]Department of Pharmaceutical Chemistry, Faculty of Pharmacy, Medical University - Sofia, Bulgaria

[2]Department of Pharmaceutical Chemistry, Faculty of Pharmacy, Suleyman Demirel University, Isparta 32260, Turkey

[3]Department of Chemistry, Government College University Faisalabad, Faisalabad 38000, Pakistan

[4]Department of Pharmacology, Pharmacotherapy and Toxicology, Faculty of Pharmacy, Medical University - Sofia, Bulgaria

[5]Department of Chemistry, Faculty of Pharmacy, Medical University - Sofia, Bulgaria

[*] Corresponding author: **e.mateev@pharmfac.mu-sofia.bg**

Table of content:

1. ^{1}H-NMR spectras
2. IR-spectroscopic spectras
3. HPLC spectra
4. Molecular docking
5. Supplementary Table 1. Bond lengths and bond angles of heteroatom-consisting parts of **vh0** before and after the optimization

1. ^{1}H-NMR spectra

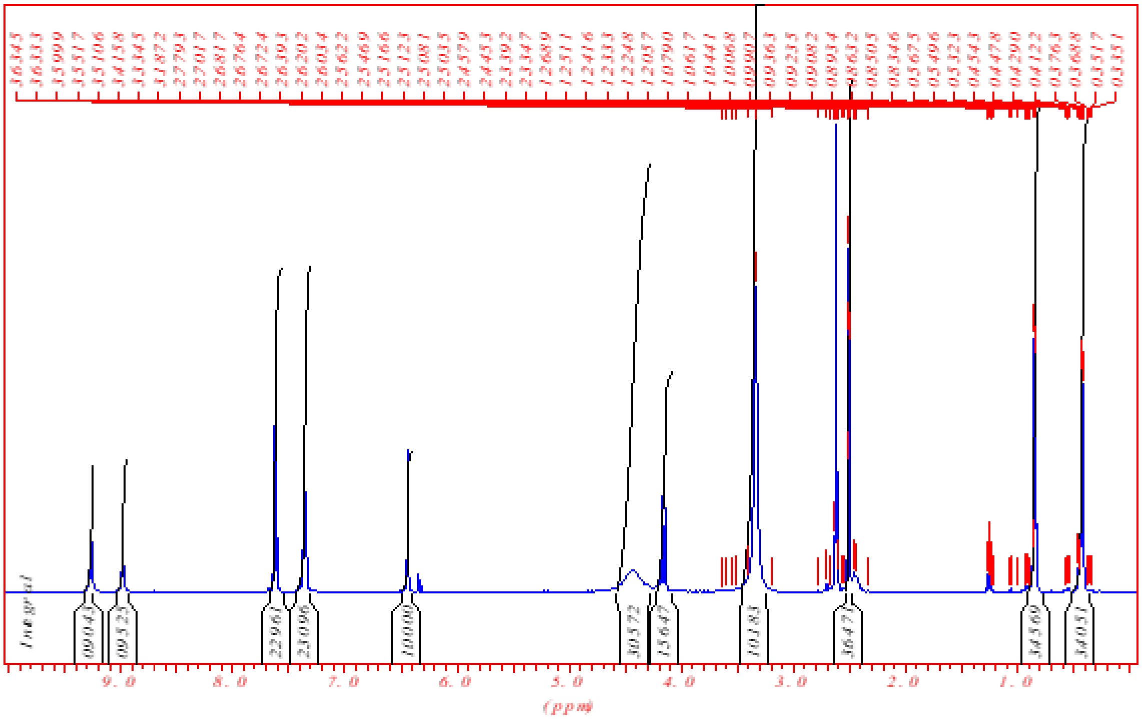

Figure S1. ^{1}H-NMR spectra of compound **vh0**.

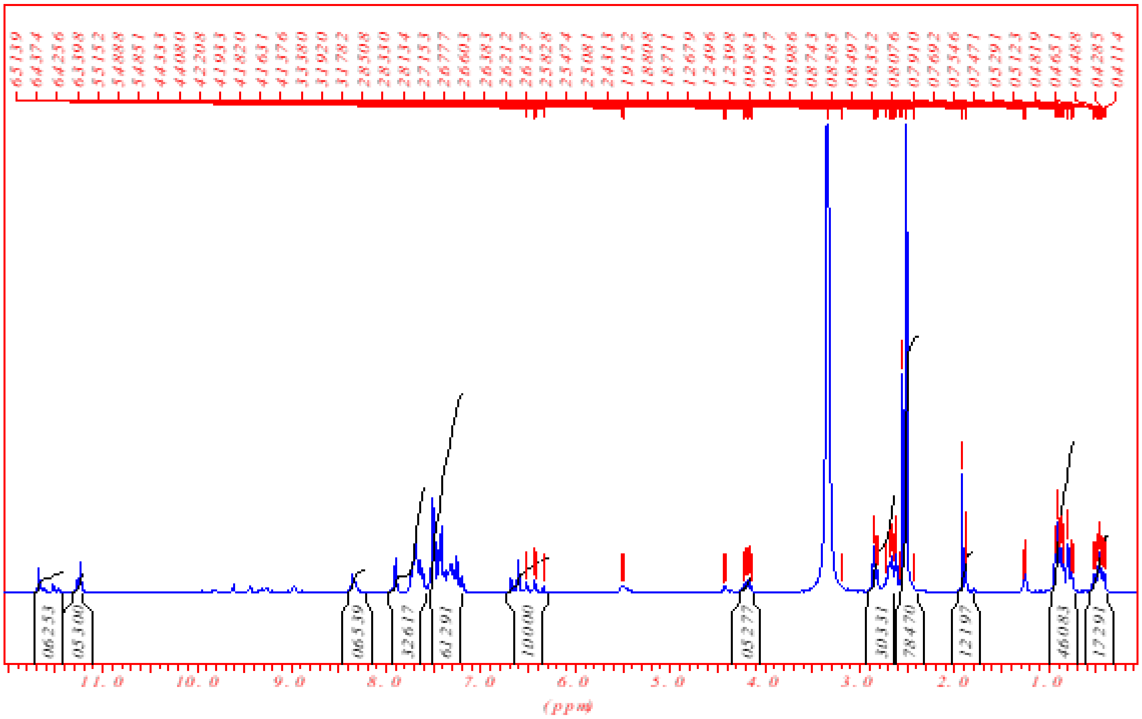

Figure S2. ^{1}H-NMR spectra of compound **vh1**.

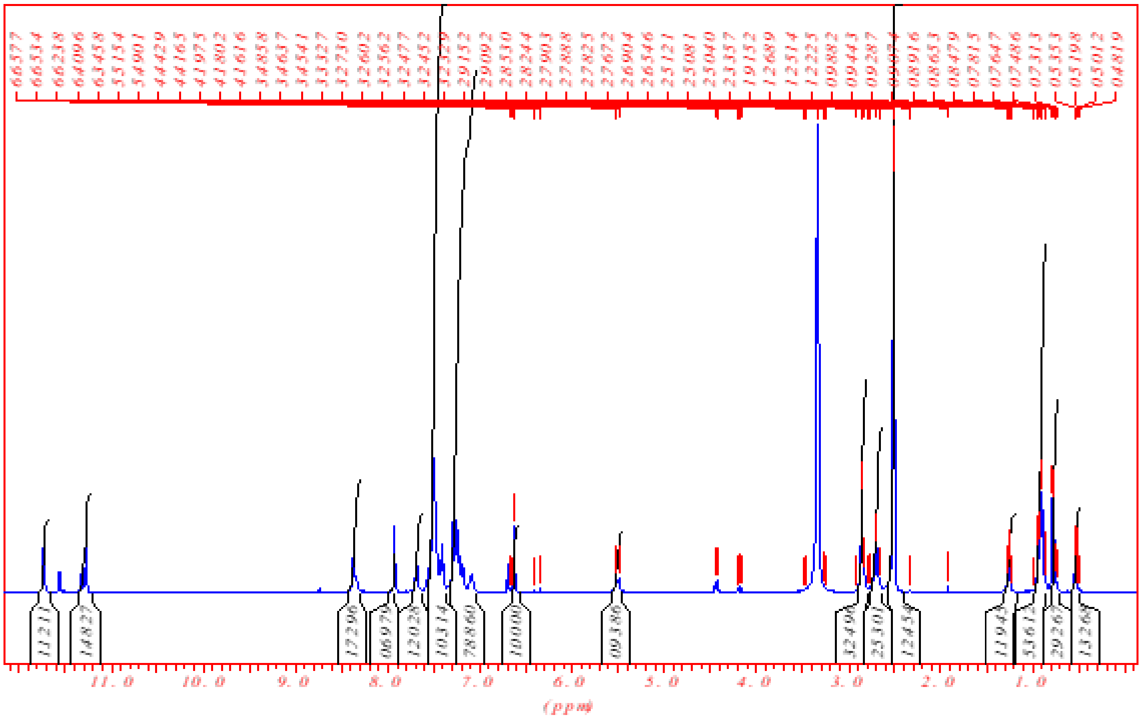

Figure S3. ^{1}H-NMR spectra of compound **vh2**.

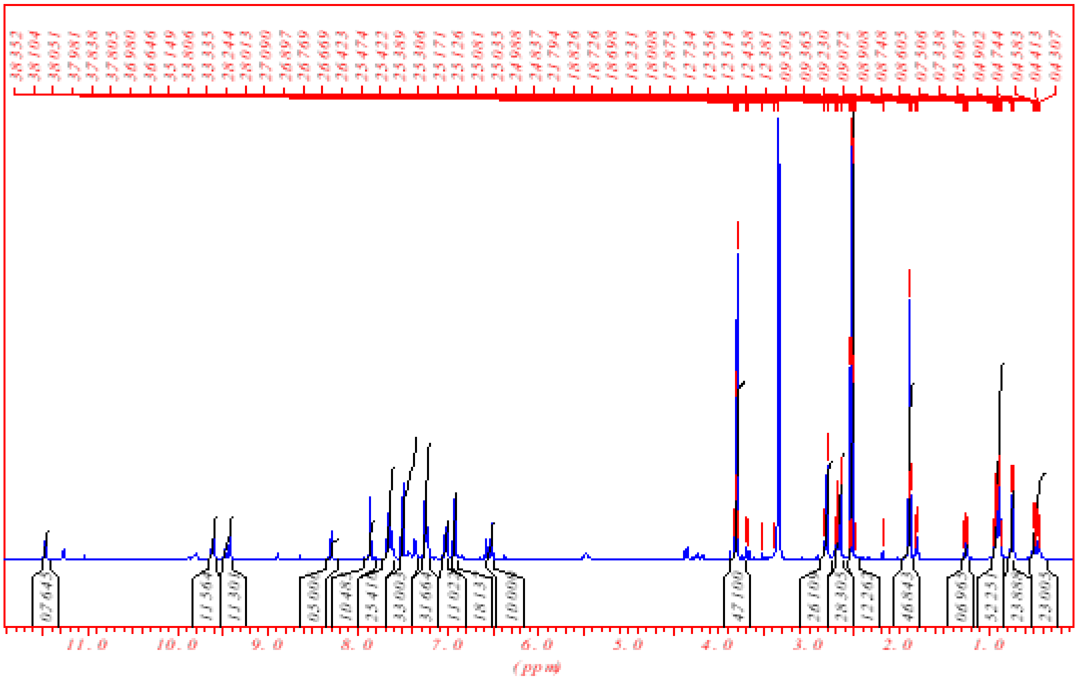

Figure S4. ^{1}H-NMR spectra of compound **vh3**.

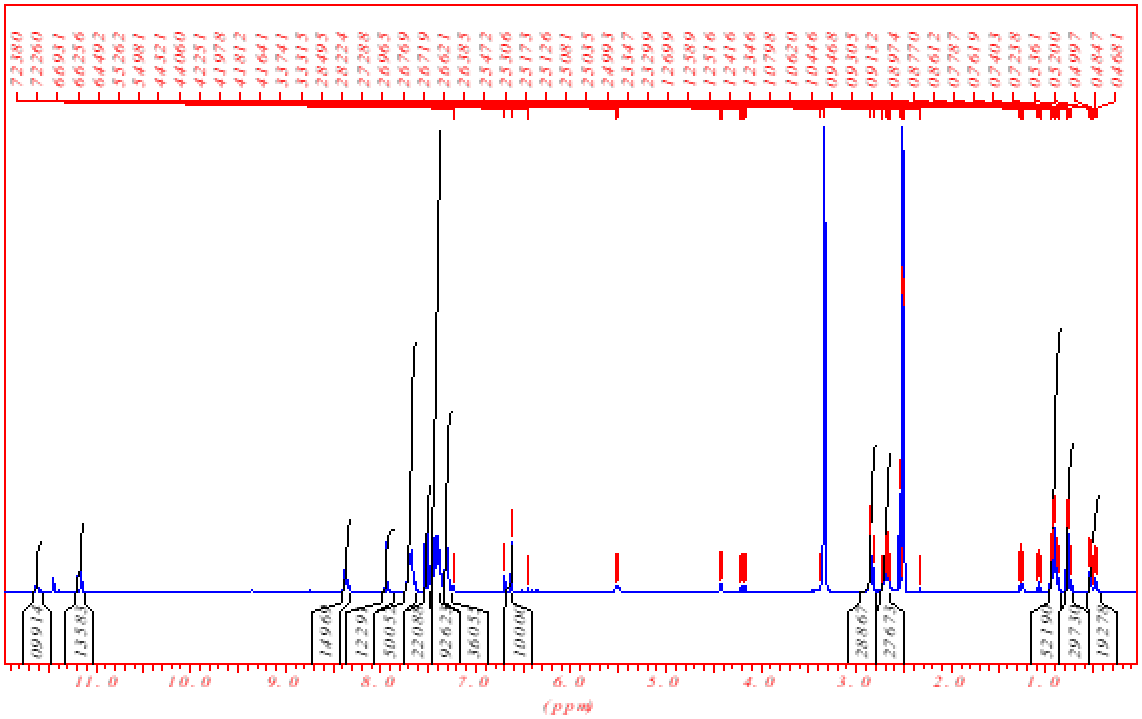

Figure S5. ^{1}H-NMR spectra of compound **vh4**.

2. IR-spectroscopic spectra

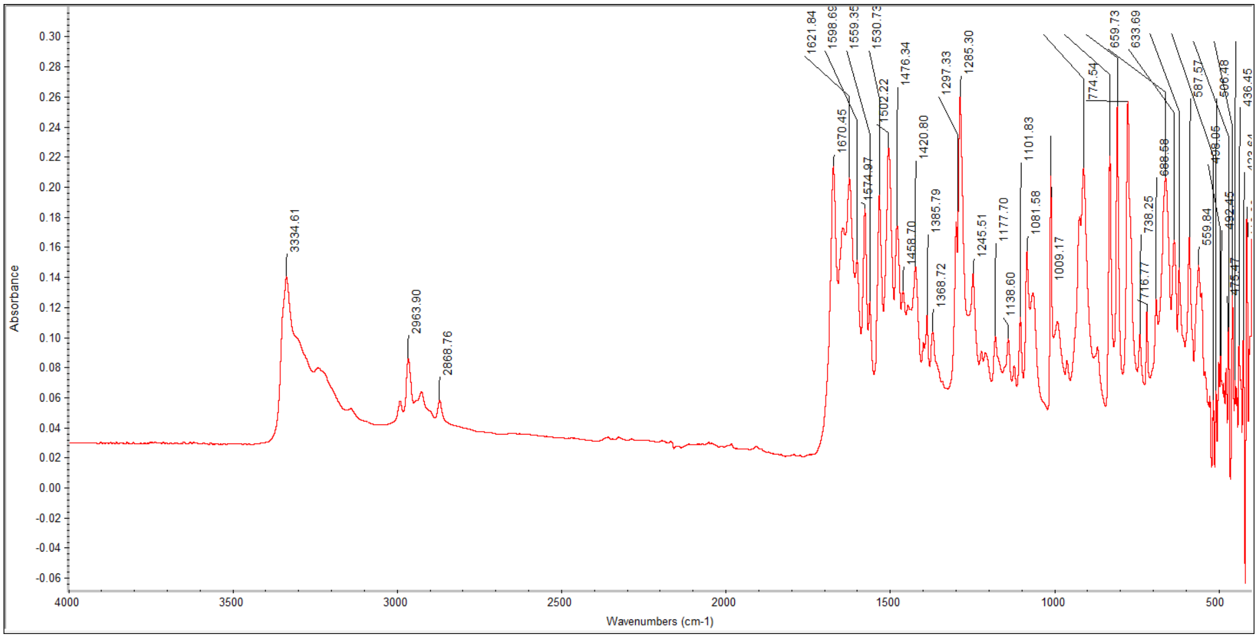

Figure S6. IR spectra of compound **vh0**.

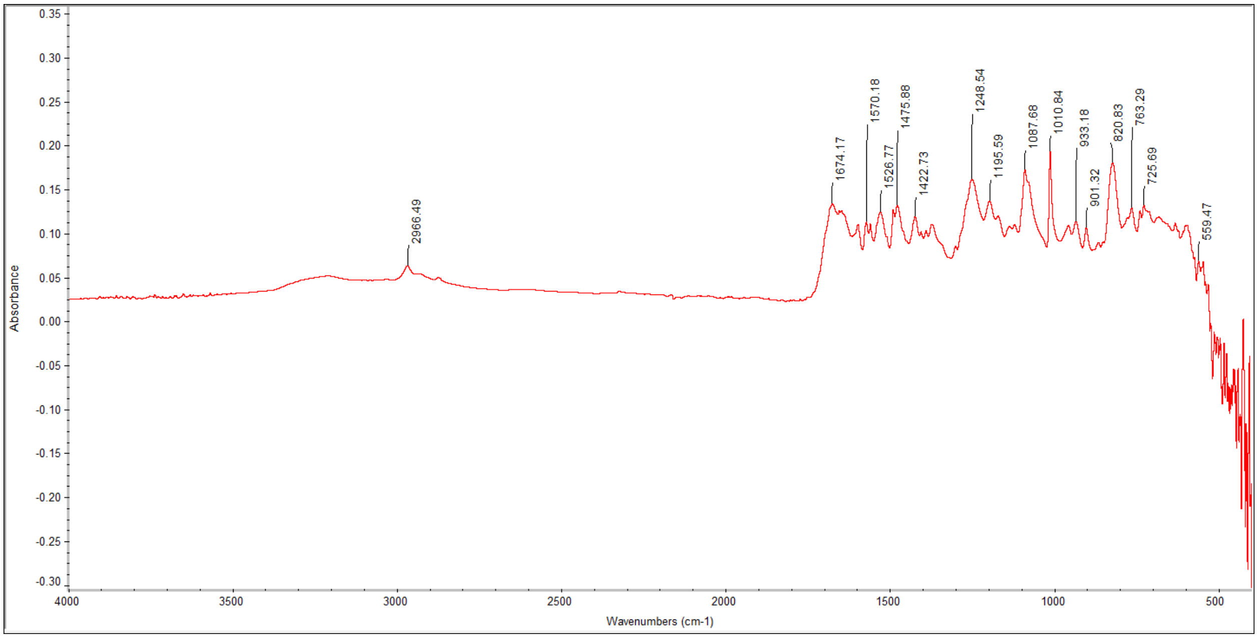

Figure S7. IR spectra of compound **vh1**.

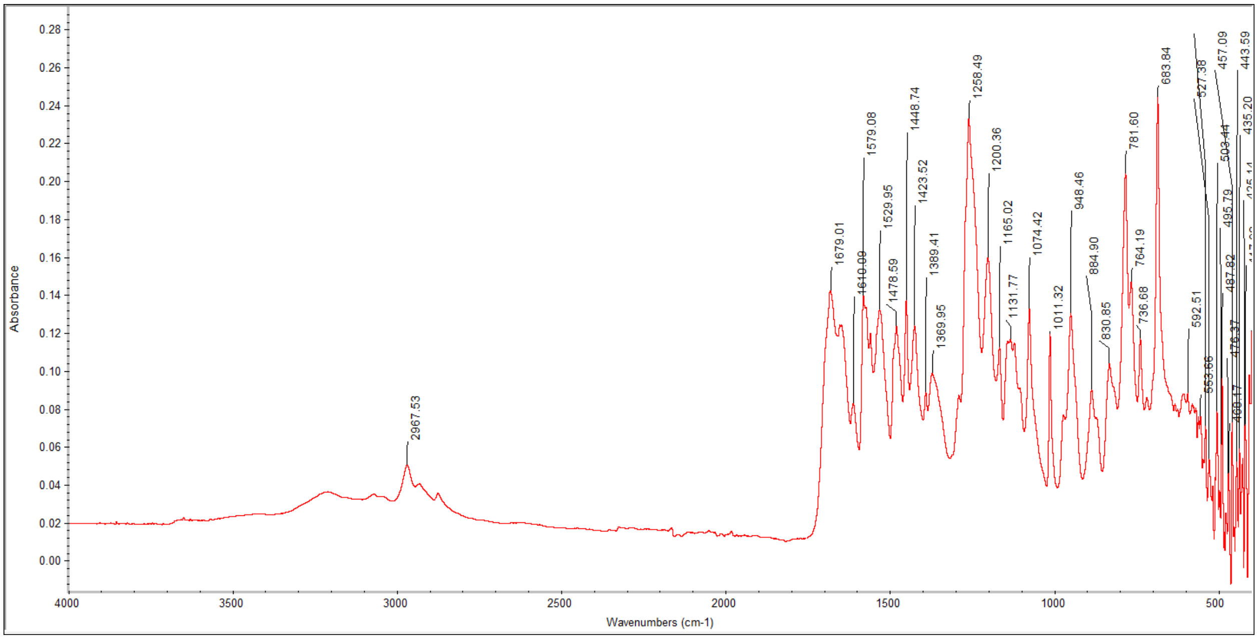

Figure S8. IR spectra of compound **vh2**.

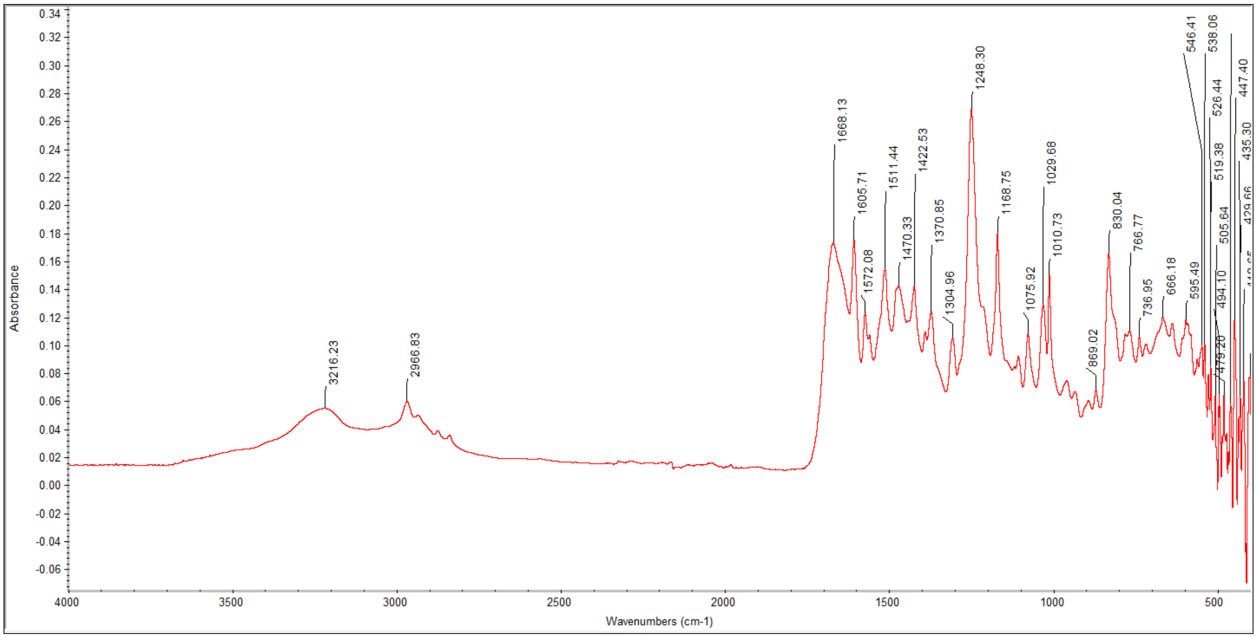

Figure S9. IR spectra of compound **vh3**.

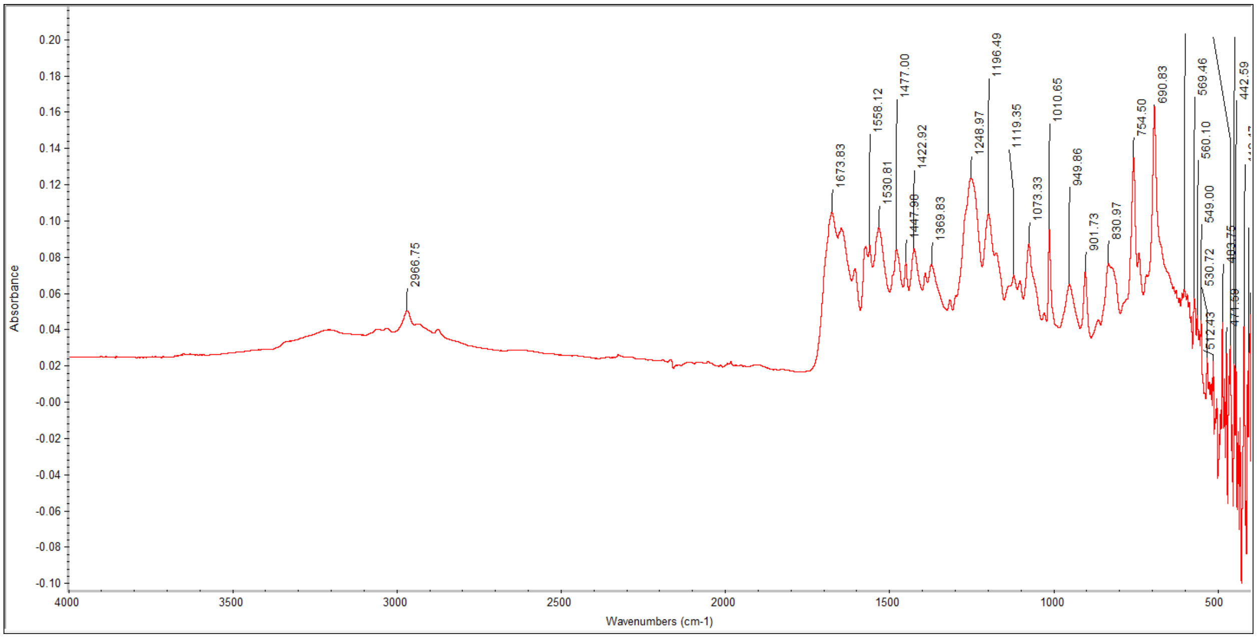

Figure S10. IR spectra of compound **vh4**.

3. HPLC spectra

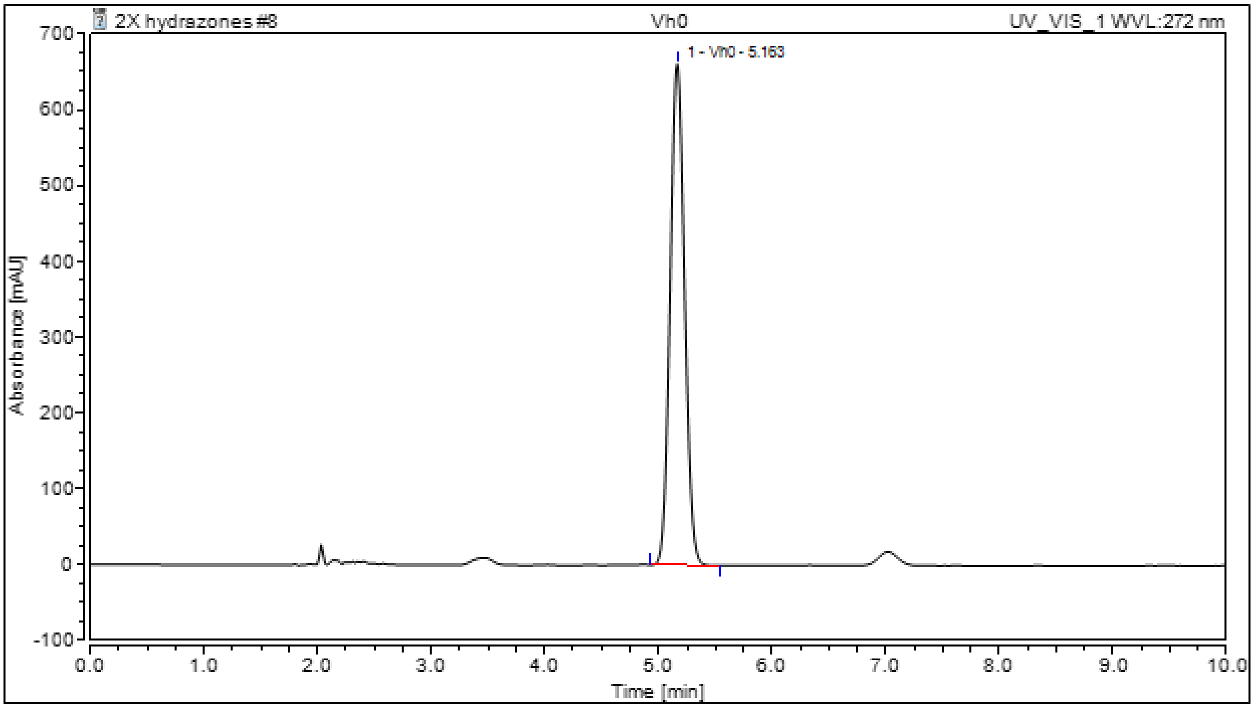

Figure S11. HPLC spectra of compound **vh0.**

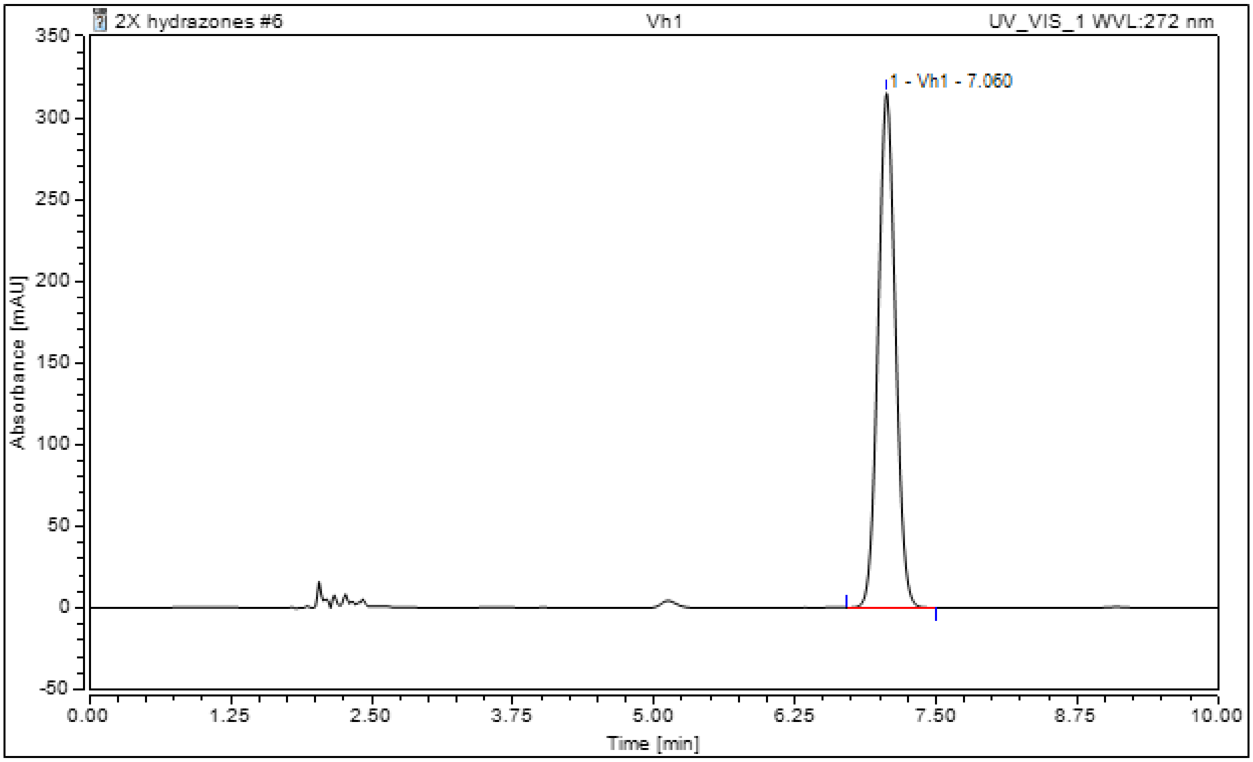

Figure S12. HPLC spectra of compound **vh1.**

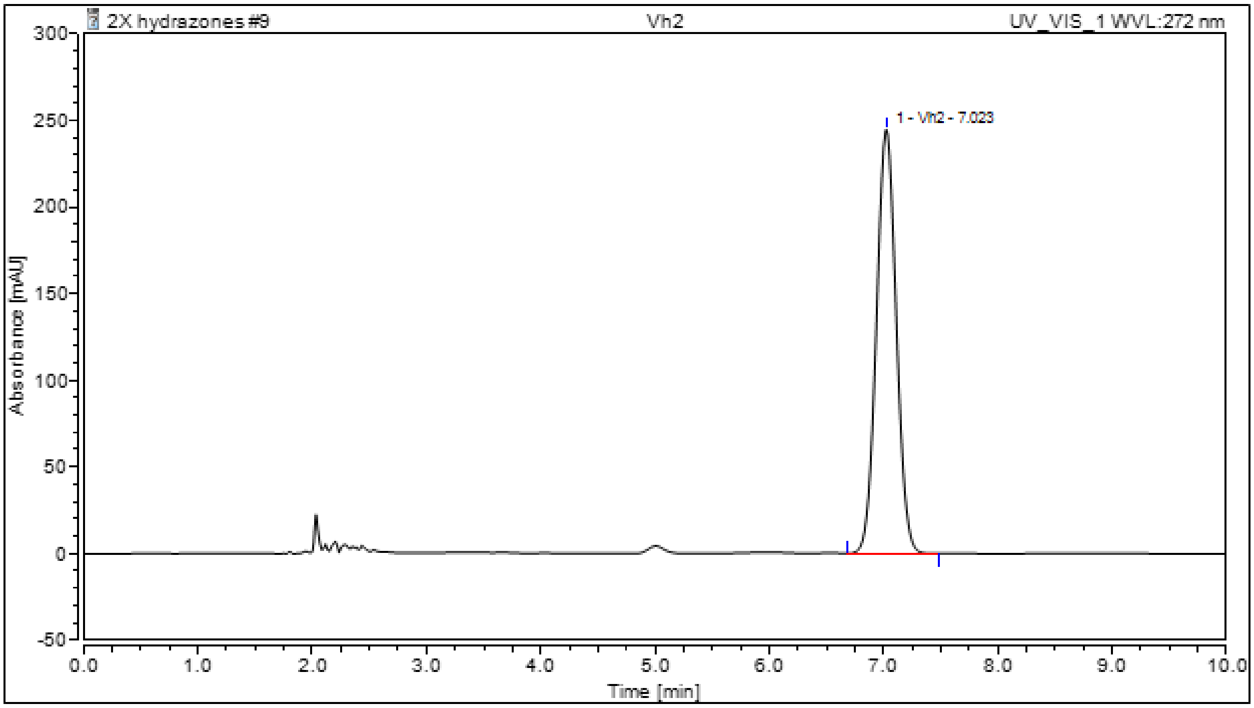

Figure S13. HPLC spectra of compound **vh2.**

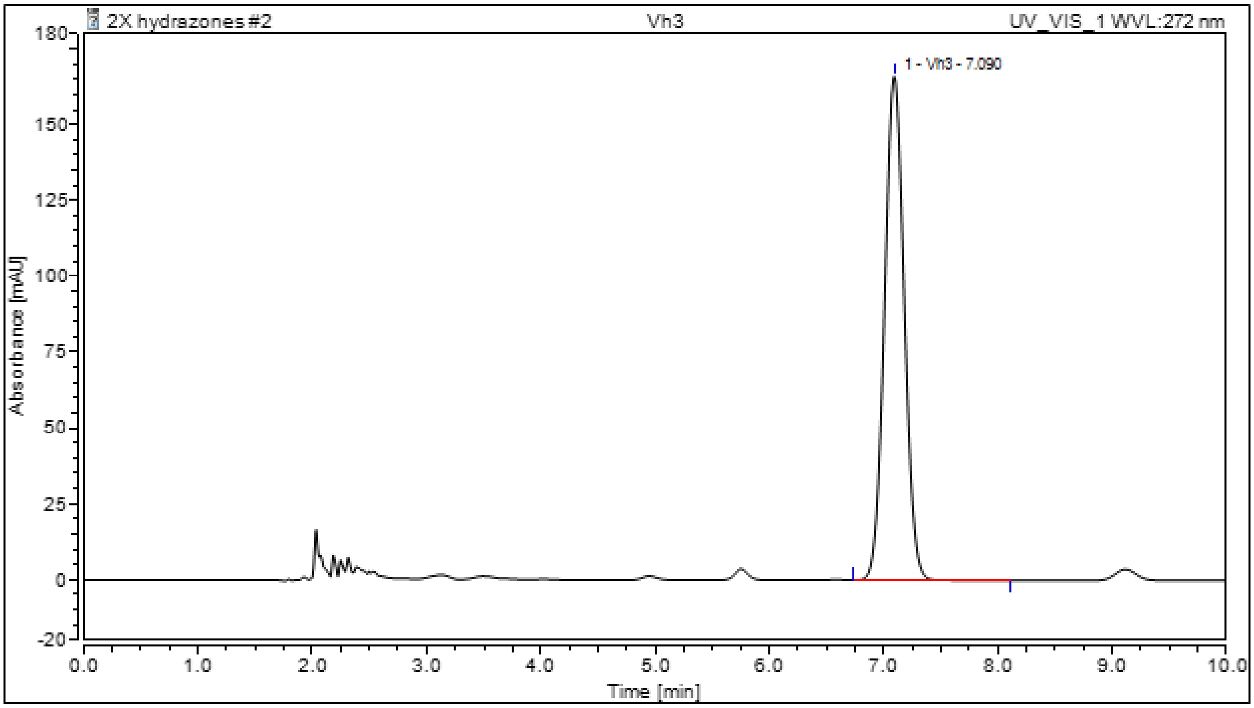

Figure S14. HPLC spectra of compound **vh3.**

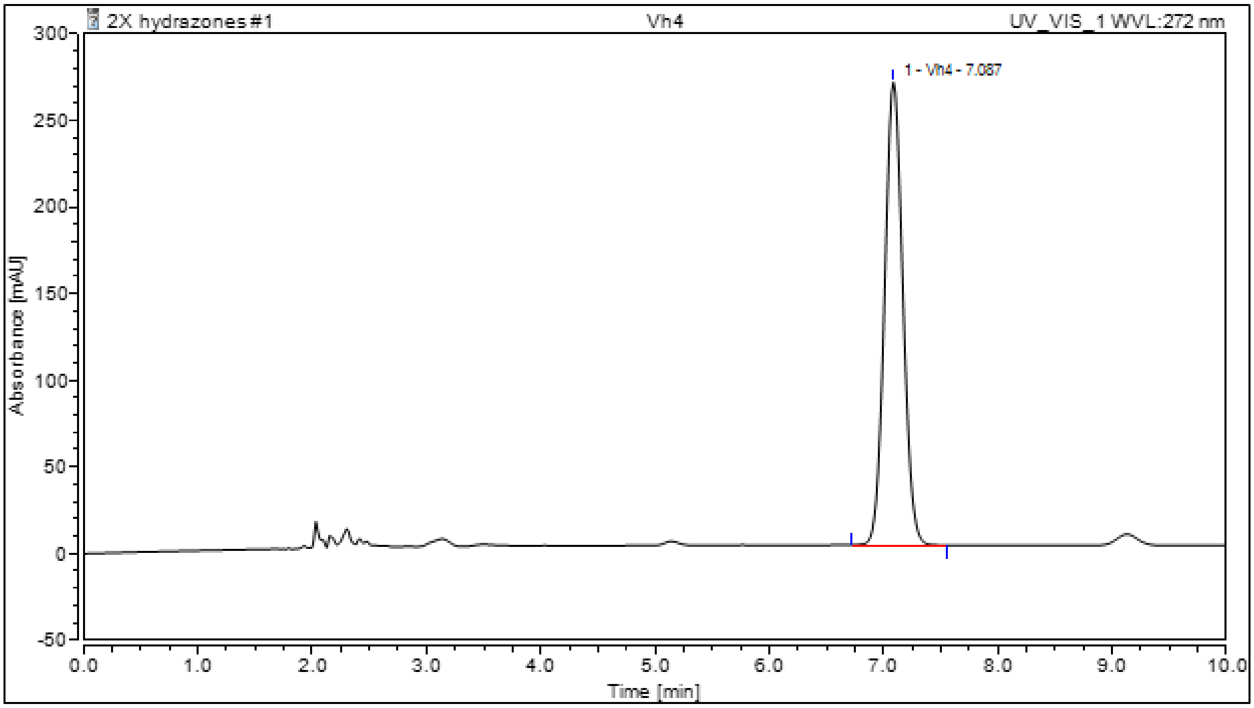

Figure S15. HPLC spectra of compound **vh4.**

4. Molecular docking studies

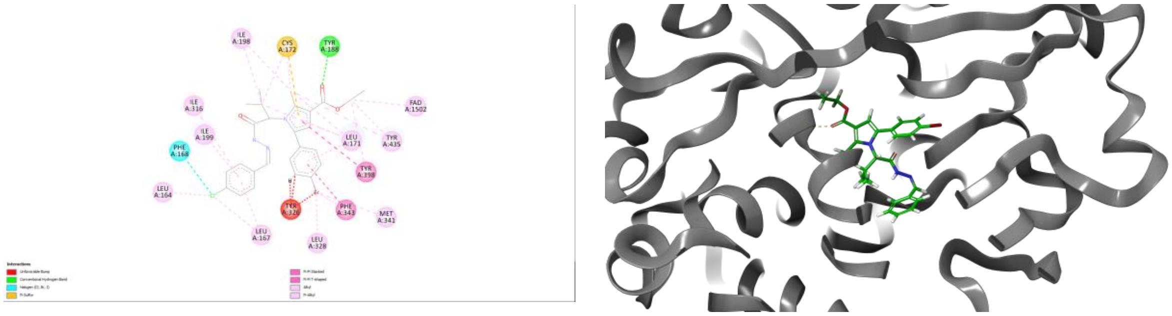

A

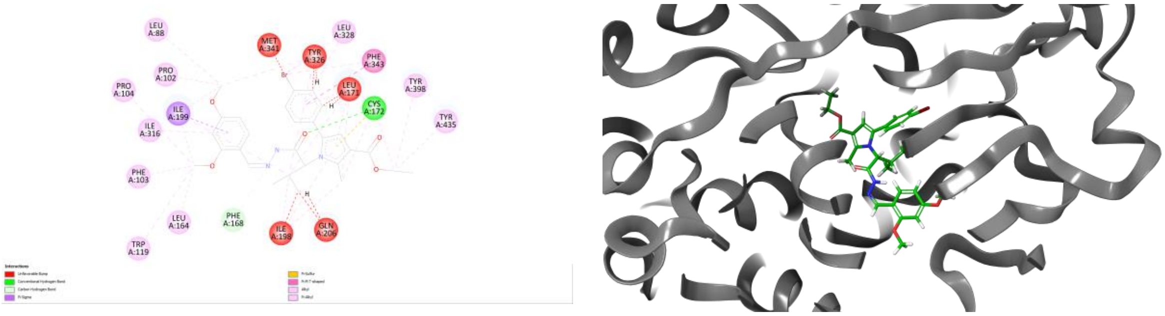

B

Figure S16. 2D and 3D interaction diagrams demonstrating the major intermolecular interactions of **vh1** (A) and **vh4** (B) with the active site of MAO-B (PDB: **2V5Z**).

Table S1. Bond lengths and bond angles of heteroatom-consisting parts of **vh0** before and after the optimization

Bond	Before Optimization (Å)	After Optimization (Å)	Bond Angle	Before Optimization (°)	After Optimization (°)
Br26-C23	1.9034	1.9493	Br26-C23-C24	119.6926	119.4930
N20-N19	1.3945	1.3998	Br26-C23-C22	119.7460	119.5901
N19-C17	1.3483	1.3618	O9-C10-C11	109.3697	106.8787
O18-C17	1.2339	1.2316	C7-O9-C10	120.8413	119.8903
C13-N1	1.4845	1.5568	C13-C17-O18	120.4553	122.9431
C10-O9	1.4215	1.5188	C13-C17-N19	115.2286	115.3677
O9-C7	1.3558	1.4592	N1-C13-C14	114.4720	113.4433
O8-C7	1.2329	1.3526	N1-C13-C17	114.0883	111.9018
C5-N1	1.3892	1.4147	C3-C7-O8	124.0190	125.7615
C2-N1	1.4002	1.3822	C3-C7-O9	111.3103	112.6416
			O8-C7-O9	124.6695	121.5969
			C6-C5-N1	126.2559	124.1786

	C12-C2-N1	123.3203	125.2806
	C3-C2-N1	107.9123	107.4397
	C13-N1-C5	123.2586	122.2484
	C13-N1-C2	128.1275	128.1032
	C5-N1-C2	108.4353	108.7989
